# ELFN1-AS1 promotes GDF15-mediated immune escape of colorectal cancer from NK cells by facilitating GCN5 and SND1 association

**DOI:** 10.1007/s12672-023-00675-6

**Published:** 2023-05-06

**Authors:** Bin Han, Jinsong He, Qing Chen, Min Yuan, Xi Zeng, Yuanting Li, Yan Zeng, Meibo He, Qilin Zhou, Dan Feng, Daiyuan Ma

**Affiliations:** 1 GCP Center/Institute of Drug Clinical Trials, Affiliated Hospital of North Sichuan Medical College, Nanchong, China; 2 Department of Pharmacy, Affiliated Hospital of North Sichuan Medical College, Nanchong, China; 3 Institute of Pharmacy, North Sichuan Medical College, Nanchong, China; 4 Department of Gastroenterology, Affiliated Hospital of North Sichuan Medical College, Nanchong, China; 5 Department of Oncology, Affiliated Hospital of North Sichuan Medical College, Nanchong, China

**Keywords:** Nature killer cell, ELFN1-AS1, GDF15, SND1, Colorectal cancer, GCN5

## Abstract

The ability of colorectal cancer (CRC) cells to escape from natural killer (NK) cell immune surveillance leads to anti-tumor treatment failure. The long non-coding RNA (lncRNA) ELFN1-AS1 is aberrantly expressed in multiple tumors suggesting a role as an oncogene in cancer development. However, whether ELFN1-AS1 regulates immune surveillance in CRC is unclear. Here, we determined that ELFN1-AS1 enhanced the ability of CRC cells to escape from NK cell surveillance in vitro and in vivo. In addition, we confirmed that ELFN1-AS1 in CRC cells attenuated the activity of NK cell by down-regulating NKG2D and GZMB via the GDF15/JNK pathway. Furthermore, mechanistic investigations demonstrated that ELFN1-AS1 enhanced the interaction between the GCN5 and SND1 protein and this influenced H3k9ac enrichment at the GDF15 promotor to stimulate GDF15 production in CRC cells. Taken together, our findings indicate that ELFN1-AS1 in CRC cells suppresses NK cell cytotoxicity and ELFN1-AS1 is a potential therapeutic target for CRC.

**Supplementary Information:**

The online version contains supplementary material available at 10.1007/s12672-023-00675-6.

## Introduction

Colorectal cancer (CRC) represents a prevalent form of malignant tumors within the digestive system and poses a substantial burden on global public health, particularly in China, where its incidence and mortality rates are notably high [1, 2]. Metastases makes this disease difficult to control and leads to a low five-year survival rate [3]. Actually, before distant metastasis occurs, tumor cells must escape from immune surveillance within the human body [4], but the underlying mechanism of immune escape in CRC remains unclear.

Increasing evidence has indicated that Long non-coding RNAs (lncRNAs) [5] regulate cancer metastasis-related signaling pathways via interacting with microRNA, mRNA and proteins, such as HOTAIR [6], AC020978 [7] and TMEM220-AS1 [8]. This highlights the dual nature of lncRNAs with both tumor suppressor and oncogene functions in cancers. For example, lncRNA LINC01569 functioned as a competing endogenous RNA (ceRNA) that competes with RAP2A for miR-381-3p binding, resulting in a complex that affected metastasis of CRC cancer cells [9]. Conversely, LINC00675 interacted with vimentin and enhanced its phosphorylation of Ser83 that resulted in a reduction of gastric cancer cell metastasis [10]. In addition, many lncRNAs are also involved in cancer cell avoidance of immune detection although the underlying mechanisms are still unclear.

The innate immune response represents the initial barrier utilized by the body to eradicate malignant cells. Among innate immune cells, natural killer (NK) cells are considered the most potent cytotoxic effectors [11, 12]. However, in cancer tissues, NK cell activity is inhibited and the insufficient infiltration of NK cells is correlated with the survival of patients with cancers [13]. lncRNAs also affect the ability of cancer cells to escape from immune surveillance by NK cells. For instance, lncRNA GAS5 has been shown to augment the cytotoxic capabilities of NK cells towards liver cancer cells through the regulation of the miR-544/RUNX3 axis [14]. In CRC, PTTG3P overexpression facilitated M2 macrophage polarization and low-expressed PTTG3P altered the infiltration of NK, CD8 + T and TFH cells [15].

ELFN1-AS1 (ELFN1 antisense RNA 1, also named MYCLo-2), is a newly discovered antisense lncRNA of ELFN1 that has a reported role as an oncogene in various solid tumors including esophageal and ovarian cancers as well as CRC [16–18]. ELFN1-AS1 is regulated by MYC and has a role in the tumorigenesis and transformation of cancer [19]. In CRC, ELFN1-AS1 has been identified as a promoter of colon cancer progression through the modulation of the miR-4644/Trim44 axis [20] and also facilitates cell invasion, migration and proliferation by sponging miR-1250 to upregulate MTA1 [21]. However, whether ELFN1-AS1 is required for the escape of CRC from immune surveillance remains unknown. In the present study, our findings indicated that ELFN1-AS1 serves a suppressor role in NK-cell surveillance and we revealed the underlying mechanism by which ELFN1-AS1 contributes to NK cell inaction.

## Materials and methods

Comprehensive details and methodologies pertaining to various conventional molecular biological experiments and bioinformatics analyses can be found in the supplementary information.

### Blood samples collection and NK cells isolation

Fresh peripheral blood samples (10) were obtained from healthy volunteers with an average age of 25.5 years. The study was approved by the Ethics Committee of the Affiliated Hospital of North Sichuan Medical College. All of the operation processes were in accordance with The Code of Ethics of the World Medical Association. Normal NK cells in peripheral blood were isolated and expanded using a Human NK Cell Enrichment Set-DM (BD Biosciences, USA) and an NK Cell Robust Expansion kit (Stemery, China) according to the manufacturer’s instructions. The purity and amplification of NK cells were identified using flow cytometry. The operations were performed as described in reference [22].

### Plasmids, primers and shRNAs

Lentiviral particles containing (i) the sequence of ELFN1-AS1 (termed Lv-ELFN1-AS1) or empty vector (termed Lv-NC), (ii) short hairpin RNAs (termed sh-RNAs) or their scrambled control, a nontargeting RNA sequence (termed sh-NC) were all designed, constructed, amplified and purified by Sangon Biotech. Detailed information about the plasmids construction and transfection was described in supplementary. Primers and shRNA sequences are listed in Table S1.

### Cell co-culture

The CRC cells were co-cultured with the NK cells at a ratio of 1: 10 for 12 h; the supernatants of conditioned CRC cells were co-cultured with NK cells for 24 h. The operations were performed as described in reference [22].

### Detection of cell proliferation, apoptosis and NK cell surface markers

See supplementary information.

### Animal experiments

For in vivo tumor growth assays, 5 × 10^6^ HCT116 cells were collected and subcutaneously injected into the left arm pit of male nude mice (male, 5 weeks of age, 6 mice/group). Purified 5 × 10^7^ NK cells were injected via the tail vein at day 7 following tumor cell inoculation and were injected once every 5 days thereafter. Tumors were measured with calipers and calculated with the formula: Volume (mm^3^) = [width^2^ (mm^2^) × length (mm)]/2. At day 21, tumors were dissected and weighed.

For in vivo pulmonary metastasis assays, approximately 5 × 10^5^ HCT116 cells were injected into nude mice via the tail vein. Purified 5 × 10^6^ NK cells were injected via the tail vein at day 5 after tumor cell inoculation and the NK cell injections were repeated every 5 days. After 4 weeks, the mice were sacrificed. The lungs were fixed in 4% paraformaldehyde and stained with H&E. Pulmonary metastasis were counted and quantified in a random selection of high-power fields. The animal experiments were performed as described previously [22]. All animal studies were approved by the Medical Experimental Animal Care Commission of Affiliated Hospital of North Sichuan Medical College and were in accordance with the National Research Council's Guide for the Care and Use of Laboratory Animals.

### Signal pathway array

Total protein of CRC cells in ELFN1-AS1- silenced cells and controls was extracted using RIPA lysis buffer containing 1% PMSF and used for signal pathway array analysis conducted by Shanghai Univ-bio Biotechnology, Art. No: ARY003B.

### Blockade of signaling

NK cells were treated with a chemical inhibitor (DB07268, 9 nM, 2 h) or DSMO (Beyotime, China), then cultured with the supernatants derived from HCT116 or HT29 cells to detect the NK cell surface markers or co-cultured with HCT116 or HT29 cells to detect the apoptosis of CRC cells.

### Antibody treatment

To explore the underlying mechanisms, NK cells were co-cultured with supernatants containing anti-GDF15 antibody or controls from CRC cells to detect the NK cell surface markers, or co-cultured with anti-GDF15 antibody pre-treated HCT116 or HT29 cells to measure the apoptosis of CRC cells.

### Co-immunoprecipitation (Co-IP) and RNA binding protein immunoprecipitation (RIP)

1 × 10^6^ CRC cells were seeded in 6-well plates and incubated at 37 °C with 5% CO_2_ for 24 h and used to generate an IP lysate containing 10% PMSF after incubation for 30 min at 4 °C. The supernatant was collected and antibodies were added as follows: for Co-IP, anti- GCN5 and SND1; for RIP, anti- SND1. The cells were shaken and incubated at 4 °C overnight and magnetic beads coupled to protein A and G were then added and incubated at 4 °C for 2 h. The protein precipitates were subjected to three washes with wash buffer, after which they were resuspended in loading buffer and subsequently boiled for 10 min. The captured proteins were separated by SDS-PAGE and then subjected to Western blotting. For RT-PCR arrays, the RNA immunoprecipited by protein A/G beads were extracted by Trizol (Beyotime, China) followed by RT-PCR to detect ELFN1-AS1.

### In situ hybridization and immunofluorescence

See supplementary information.

### Detecting the interaction between exogenous GCN5 and SND1

5 × 10^5^ HCT116 cells with ELFN1-AS1 silence or not were inoculated into 6-well plate and cultured for 24 h at 37 °C and 5% CO_2_. The plasmids with Flag-GCN5 and HA-SND1 were co-transfected into CRC cells using PEI. After 48 h, we added the immunoprecipitation (IP) lysate containing 10% PMSF into culture plates for 30 min at 4 °C, and then collected the supernatant after centrifuging at 12,000*g*. 10 μl Magnetic beads coupled with Flag or HA were added into the supernatant, and then incubated on shaking table at 4 °C for 3 h. The magnetic beads were washed three times with pre-cool wash buffer, add loading buffer and boiled for 10 min at 100 °C to detect the effect of protein IP. The pull-down proteins were separated by SDS-PAGE and were then subjected to western blotting.

### Statistical analysis

All data analysis were conducted using SPSS v18.0. Data are expressed as means ± standard errors of the mean (SEM). When data were normally distributed and had homogenous variances, the Student’s t-test was used for comparisons between two groups and one-way ANOVA followed by Dunnett’s post-hoc tests were used in comparisons between 3 or more groups; when the data violated the normality or homogeneity of variances, Mann–Whitney test followed by Tamhane's T2 test was performed in the comparisons between two groups and Kruskal–Wallis test followed by Dunnett's T3 tests was performed in the comparisons between 3 or more groups. *P* < 0.05 was considered statistically significant. Statistical analysis was performed as described previously [23].

## Results

### ELFN1-AS1 expression is frequently increased in CRC tissues and is associated with poor patient survival

We initially found a significantly upregulated lncRNA ELFN1-AS1 in CRC tissues from circlncRNAnet (Fig. S1A). ELFN1-AS1 has many alternatively spliced isoforms in different cancers (Fig. S2A). ORF Finder analysis illustrated that ELFN1-AS1 was unable to encode protein (Fig. S2B), Fig. S2C, D exhibited the sequence and secondary structure of ELFN1-AS1. Estimations of subcellular locations indicated that ELFN1-AS1 is expressed predominantly in the cytoplasm, cytosol, ribosome and exosome (Fig. S2E). Cytoplasmic/nuclear location analysis indicated that ELFN1-AS1 was localized to the cytoplasm in GM12878, HUVEC and K562 cells, whereas to the nuclear in Hela-S3 cells (Fig. S2F). Data from Annolnc2 indicated that ELFN1-AS1 was rarely expressed in normal samples (Fig. S1B) while highly expressed in colon adenocarcinoma (COAD), leukemia and ovarian serous cystadenocarcinoma (Fig. S1C). In multiple cancer cell lines, ELFN1-AS1 also exhibited a high expression in comparison to normal cell lines (Fig. S1D). Analysis from GEPIA2 revealed that ELFN1-AS1 levels were significantly upregulated in the COAD and rectum adenocarcinoma (READ) as compared with those in the normal tissues (Fig. S1E). Moreover, COAD and READ patients with high expression of ELFN1-AS1 exhibited significantly lower overall survival (OS) when compared with that of patients with low ELFN1-AS1 (Fig. S1F). But receiver operating characteristics (ROC) reflected that ELFN1-AS1 had weak prognostic performance in identifying overall survival analysis (Fig. S1G). Together, these data indicated that ELFN1-AS1 expression was frequently increased in CRC samples when compared with normal tissues and was associated with poor patient survival.

### ELFN1-AS1 promotes CRC cell escape from NK surveillance in vitro and in vivo

CRC pathogenesis and NK cells activity are closely linked [24]. GEPIA2021 results indicated that activated NK cells were present at significantly higher levels than resting ones in normal tissues, whereas significantly suppressed in COAD and READ tumor tissues (Fig. S3A). Furthermore, in COAD and READ, patients with higher numbers of NK cells showed a longer disease-free survival (DFS, P = 0.02) (Fig. S3B) and overall survival (OS, P = 0.06, close to the point of difference) (Fig. S3C), while patients with a higher number of resting NK cells exhibited a worse OS (P = 0.05) (Fig. S3D) and DFS (P = 0.06, close to the point of difference) (Fig. S3E). In tumor and normal tissues of COAD and READ, ELFN1-AS1 tended to be negatively associated with CD56 (the surface marker of NK cells) (Fig. S3F), but not significantly associated with CD16 (Fig. S3G). Based on these results, we sought to explore the relationship between NK cells and ELFN1-AS1 levels in CRC cells.

**Fig. 1:**
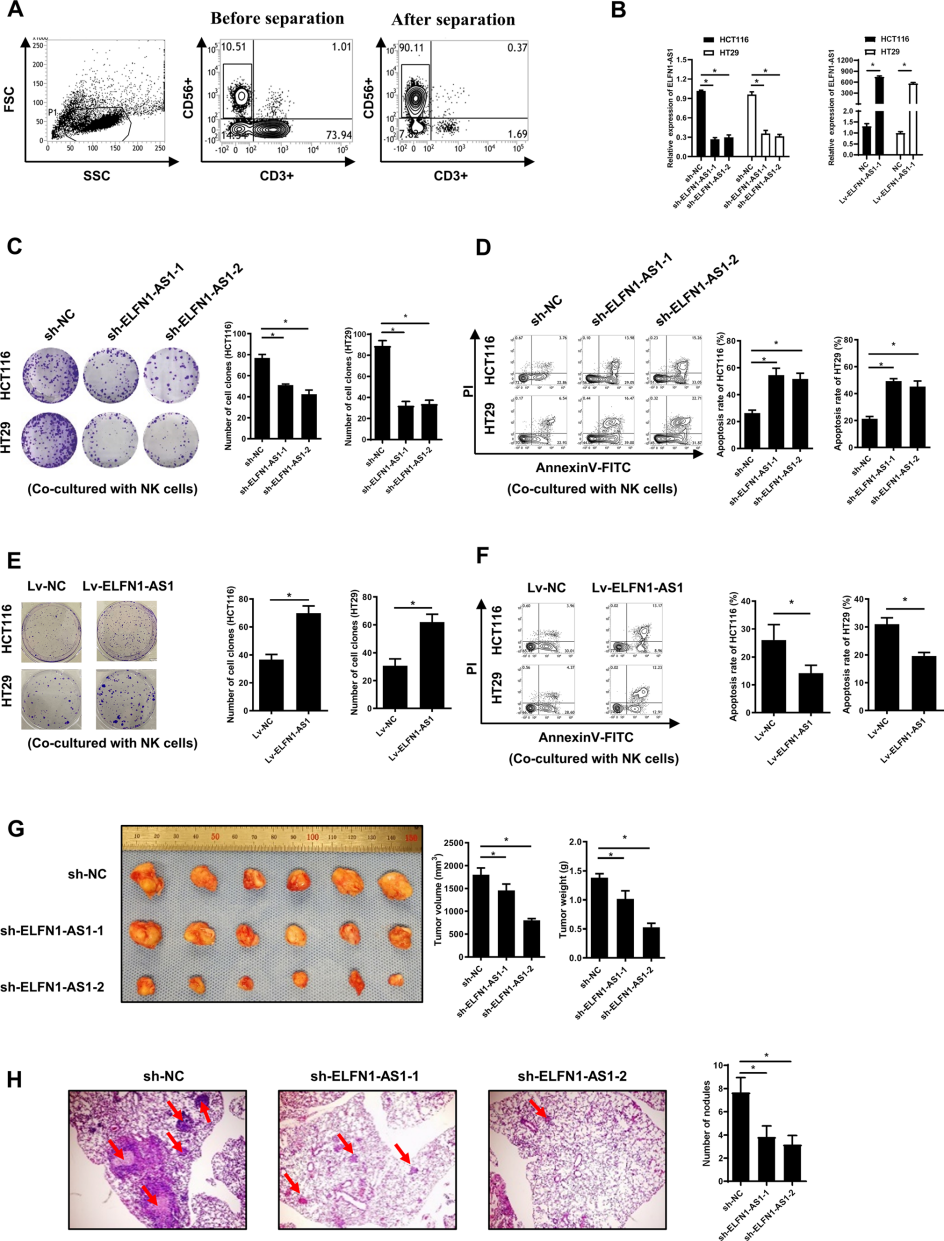
ELFN1-AS1 promotes the escape of CRC cells from NK cell surveillance in vitro and in vivo.** A** NK cells isolated from the peripheral blood by negative magnetic separation. **B** qRT-PCR was used to assess the ELFN1-AS1 expression levels in stably ELFN1-AS1-silenced or -overexpressed CRC cell lines. After co-culture with NK cells, the cell colony formation (**C**) and apoptosis (**D**) of ELFN1-AS1 knockdown CRC cell lines cell; the colony formation (**E**) and apoptosis (**F**) of ELFN1-AS1-overexpressing CRC cell lines. **G** Volumes and weights of subcutaneously xenografted CRC tissue in nude mice (n = 6). **H** Tumor metastases in the lung of nude mice. All data are from at least three independent experiments, *P < 0.05 indicated a significant difference

NK cells from the peripheral blood were isolated by negative magnetic separation (Fig. 1A) and we knocked down ELFN1-AS1 expression in HCT116 and HT29 cells using shRNA virus or transfected the lentivirus harboring ELFN1-AS1 sequence into HCT116 and HT29 cells (Fig. 1B). To determine whether ELFN1-AS1 could affect the cytotoxicity of NK cells against CRC cells, ELFN1-AS1-overexpressing or -knockdown cells were co-cultured with the isolated NK cells. Colony formation assays and flow cytometry revealed that ELFN1-AS1 knockdown significantly decreased the colony formation capacity (Fig. 1C) and increased the apoptosis of HCT116 and HT29 cells (Fig. 1D). However, more cell colonies (Fig. 1E) and a lower level of apoptosis (Fig. 1F) were observed when ELFN1-AS1 was overexpressed. Without the co-culturing with NK cells, the colony formation capacity and apoptosis of CRC cells showed no significant differences after ELFN1-AS1 overexpressed (Fig. S3H and I). To further determine whether ELFN1-AS1 could enhance the immune escape of CRC cells from NK cells in vivo, we injected ELFN1-AS1- knockdown HCT116 cells into the flanks of BALB/c nude mice for tumorigenesis or into the lateral tail vein for pulmonary metastasis. Following CRC cell injection, NK cells were injected into lateral tail vein for testing the naturally cytotoxicity. As expected, in comparison with the control cells, ELFN1-AS1-silenced HCT116 cells exhibited impaired tumorigenesis (Fig. 1G) and metastasis capacity (Fig. 1H). This demonstrated that the expression of ELFN1-AS1 in CRC cells is related to the natural cytotoxicity of NK cells against tumor cells. Taken together, these data indicated that high levels of ELFN1-AS1 promoted the immune escape of CRC cells from NK cells in vitro and in vivo.

### ELFN1-AS1 overexpression in CRC cells impairs NK cytotoxic activity by downregulating NKG2D and GZMB

**Fig. 2:**
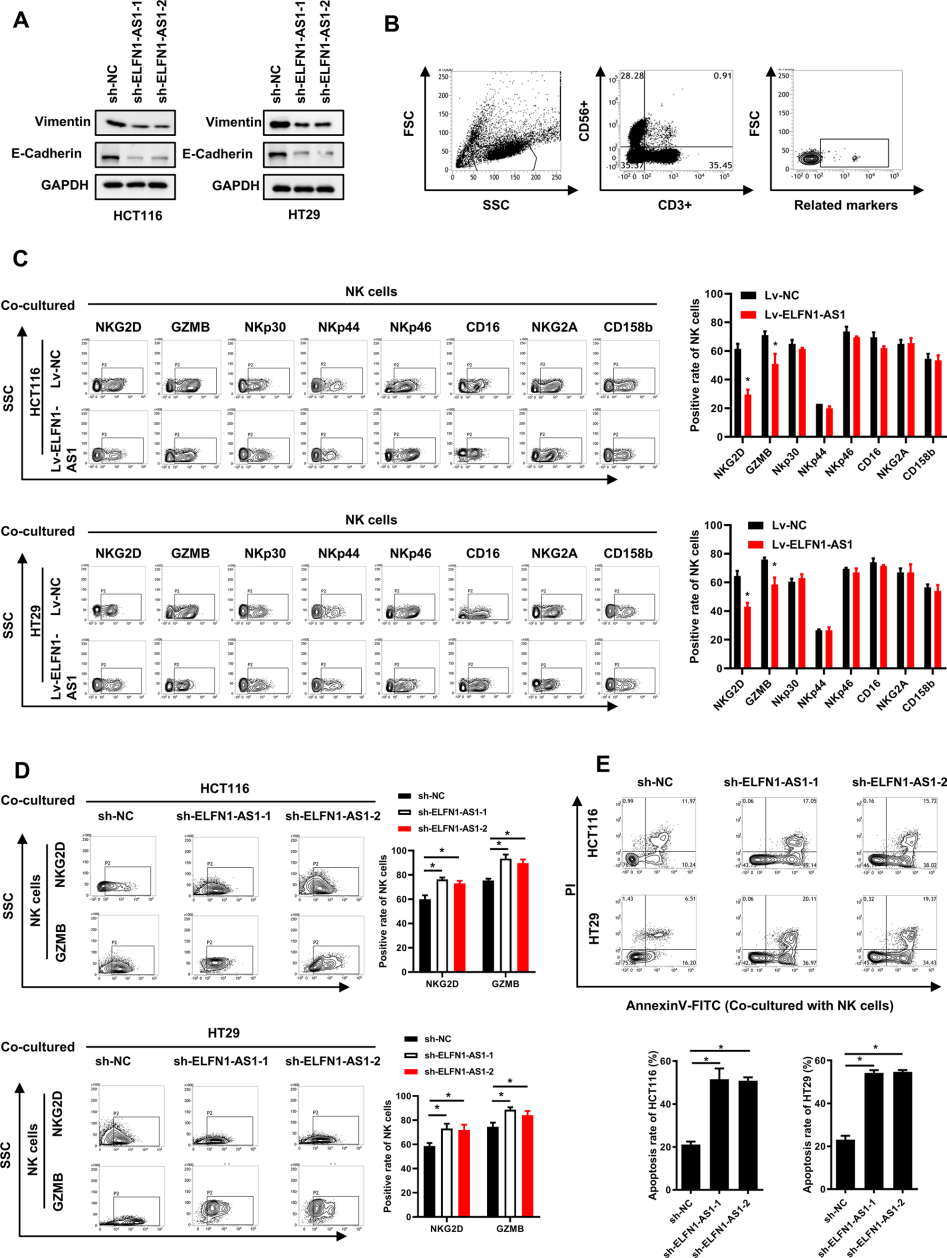
CRC cells overexpressing ELFN1-AS1 impairs NK cell cytotoxicity by downregulating NKG2D and GZMB.** A** Western blot analysis was used to assess the expression of epithelial mesenchymal transition makers in ELFN1-AS1- knockdown CRC cell lines. **B** Representative flow cytometry gates for assessing the expression of receptors in NK cells. **C** Flow cytometry was used to assess the expression of receptors in NK cells that were co-cultured with CRC cells. **D** Flow cytometry was used to assess the expression of NKG2D and GZMB in NK cells that were co-cultured with CRC cells. **E** AnnexinV-FITC/PI double-staining was used to detect the apoptosis of HCT116 and HT29 cells induced by the NK cells that were stimulated by CRC cell supernatants. All data are from at least three independent experiments, *P < 0.05 indicated a significant difference

There is evidence that ELFN1-AS1 promotes the epithelial mesenchymal transition (EMT) of multiple tumor cells [25]. In current study, we found that decreasing ELFN1-AS1 downregulated vimentin and E-cadherin in CRC cells (Fig. 2A). Tumor cells with EMT alterations are also reported to influence the activity of immune cells [26]. Here, we collected supernatants from ELFN1-AS1- overexpressing HCT116 and HT29 cells that were then added to NK cell cultures and analyzed expression of NK cell receptors using flow cytometry (Fig. 2B). The levels of activated receptor NKG2D and effector granzyme B (GZMB) were significantly decreased in the NK cells when cultured with the supernatants derived from ELFN1-AS1-overexpressing CRC cells, whereas other receptors had no significant alterations (Fig. 2C). Moreover, the supernatants derived from ELFN1-AS1-silenced CRC cells could not inhibit the appearance of NKG2D and GZMB on the surface of NK cells (Fig. 2D). NK cells stimulated by supernatants from ELFN1-AS1-silenced CRC cells had higher cytotoxicity compared to the NK cells cultured by the supernatant from control CRC cells (Fig. 2E). Taken together, these results suggested that the CRC cells with high ELFN1-AS1 may impair the cytotoxicity of NK cells by downregulating NKG2D and GZMB expression.

### ELFN1-AS1 in CRC cells downregulates NKG2D and GZMB expression in NK cells through JNK signaling

**Fig. 3:**
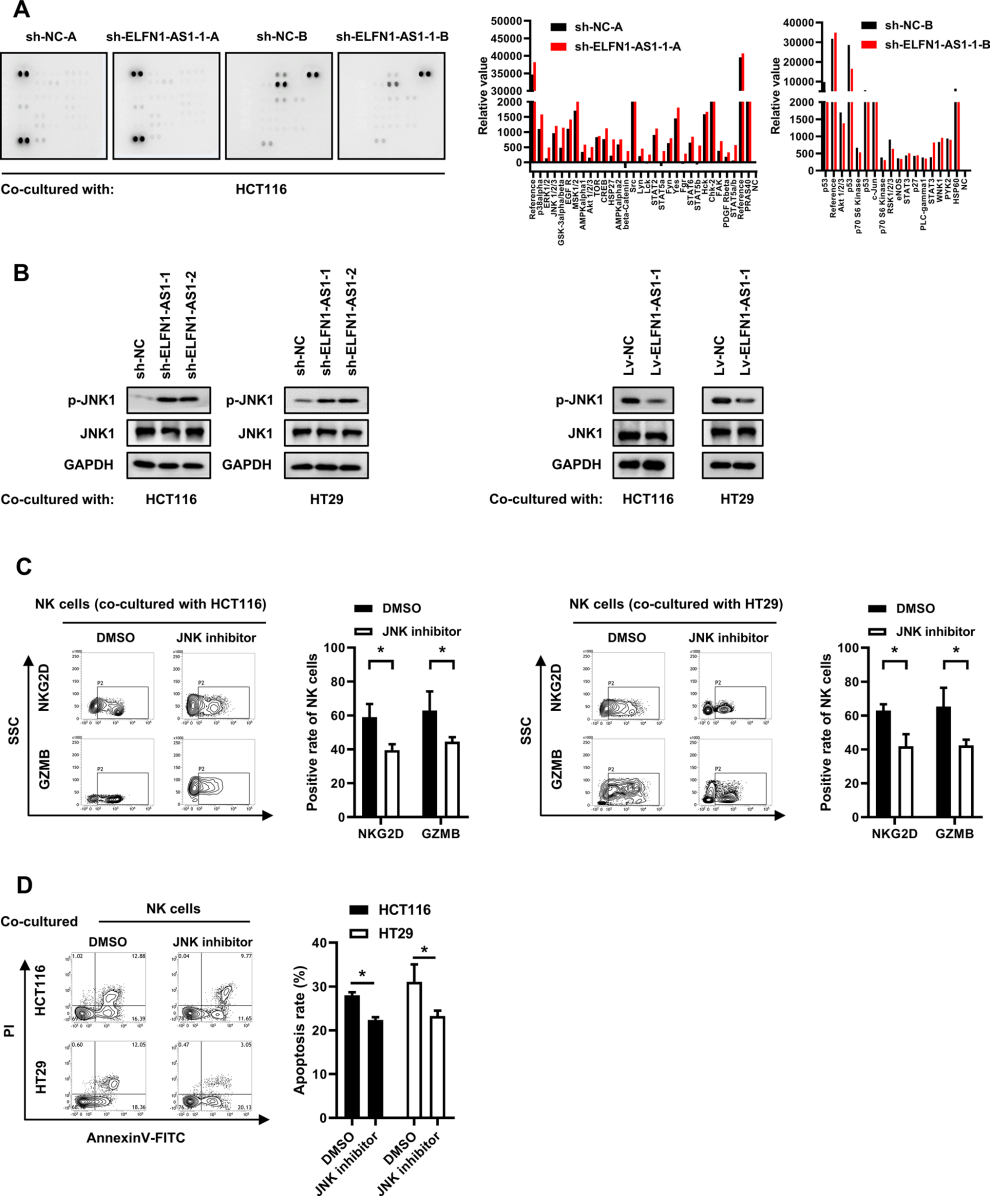
CRC cells downregulates NKG2D and GZMB expression in NK cells through JNK signaling.** A** Signal pathway array was conducted to assess the phosphorylation of several proteins in NK cells that were co-cultured with HCT116 cells. **B** Western blot analysis was used to assess the JNK signaling activity in NK cells that were co-cultured with ELFN1-AS1-overexpressing or -knockdown CRC cells. **(C)** Flow cytometry was used to assess the expression of NKG2D and GZMB in JNK signaling- blocked NK cells that were co-cultured with HCT116 or HT29 cells. **D** AnnexinV-FITC/PI double-staining was used to detect the apoptosis of HCT116 and HT29 cells induced by the NK cells treated with a JNK inhibitor. All data are from at least three independent experiments, *P < 0.05 indicated a significant difference

Pathway array elucidated that phosphorylation of several proteins in NK cells were increased after co-culture with ELFN1-AS1-silenced CRC cells (Fig. 3A). Among these, JNK signaling has been reported to regulate the expression of NKG2D [27]. As expected, JNK1 phosphorylation in NK cells could be induced by co-culture with ELFN1-AS1-silenced CRC cells and suppressed by co-culturing with CRC cells expressing high ELFN1-AS1 levels (Fig. 3B). Cultured with the supernatant from CRC cells, the ratio of NKG2D + and GZMB + NK cells were significantly decreased in the JNK pathway inhibitor pre-treated NK cells as compared to DMSO (Fig. 3C). Consistent with this, blocking JNK signaling significantly reduced the NK cell cytotoxicity against HCT116 and HT29 cells (Fig. 3D), indicating that NKG2D and GZMB expression in NK cells were primarily regulated by JNK signaling.

### ELFN1-AS1 promotes the expression and secretion of GDF15 in CRC cells to escape NK cell surveillance

**Fig. 4:**
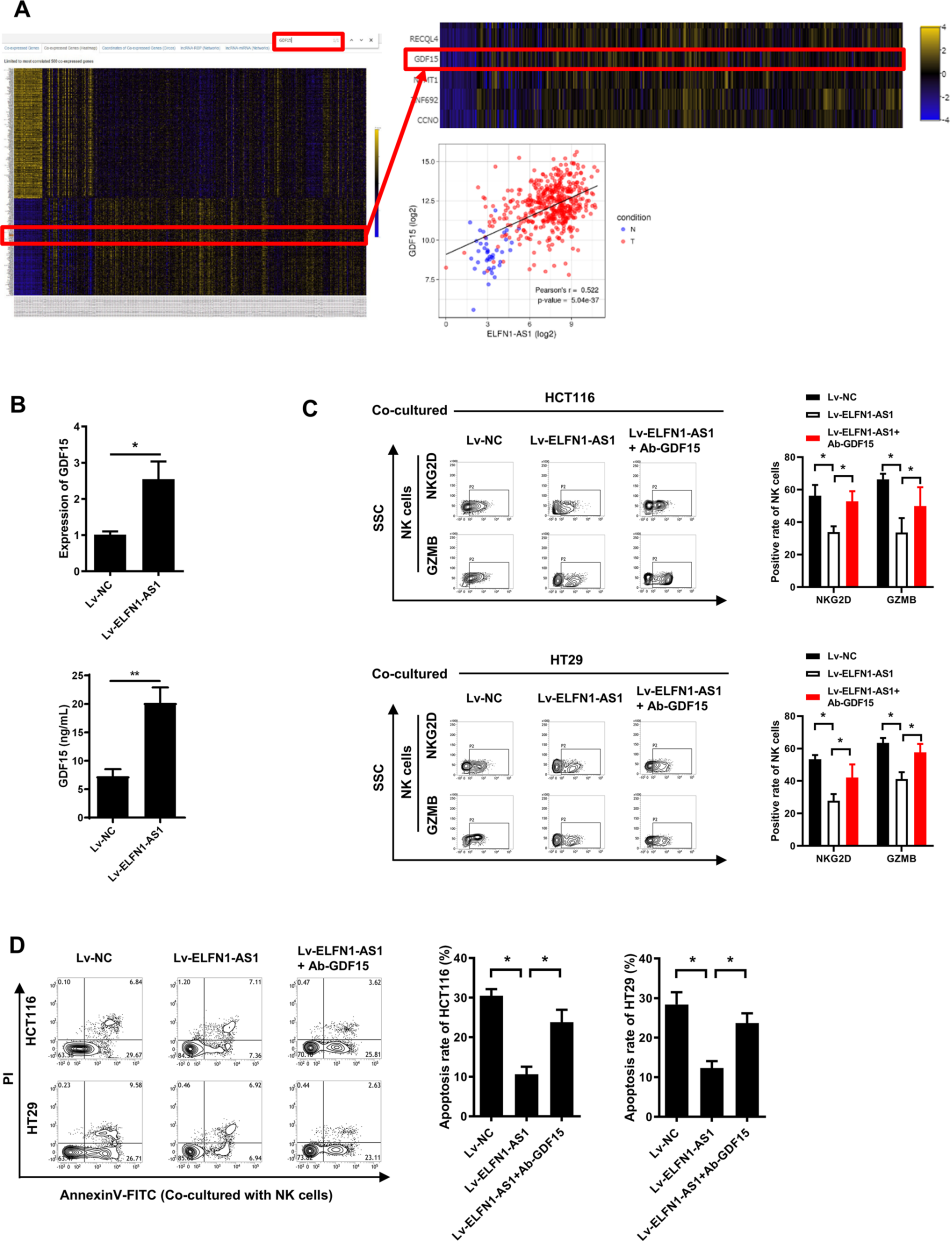
ELFN1-AS1 promotes the expression and secretion of GDF15 in CRC cells to escape from NK cell surveillance.** A** Correlation between ELFN1-AS1 and GDF15 in CRC tissues (data from circlncRNAnet). **B** qRT-PCR and Western blot was used to assess the mRNA and protein levels of GDF15 in ELFN1-AS1-overexpressing HCT116 cells. Following co-culture with CRC cells treated with an anti-GDF15 antibody, Flow cytometry was used to assess the NKG2D and GZMB expression in NK cells (**C**) and AnnexinV-FITC/PI double-staining was used to detect the apoptosis of HCT116 and HT29 cells induced by the NK cells (**D**). All data are from at least three independent experiments, *P < 0.05 indicated a significant difference

Tumor cells can impair immune cell activity through cytokine secretion such as TGF-β [28]. We found that the expression of GDF15 (a secretory ligand protein belonging to the TGF-β superfamily) was positively correlated with ELFN1-AS1 in CRC tissues (Fig. 4A). Then, ELFN1-AS1 was confirmed to promote GDF15 expression in CRC cells (Fig. 4B). To evaluate whether ELFN1-AS1 in CRC cells regulated the activity of NK cells via GDF15, we added a GDF15-specific antibody into the supernatant of ELFN1-AS1 overexpressing CRC cells and then co-cultured with NK cells. The NKG2D and GZMB expression in NK cells was suppressed by the supernatants from ELFN1-AS1 overexpressing CRC cells and was significantly restored with the addition of GDF15 antibody (Fig. 4C). Moreover, the NK cell-induced apoptosis of ELFN1-AS1-overexpressing CRC cells was significantly increased by GDF15 antibody pre-treatment (Fig. 4D). Taken together, these results indicated that ELFN1-AS1 in CRC cells promoted the immune escape of the cancer cells from NK cells by facilitating GDF15 synthesis and secretion.

### ELFN1-AS1 regulates GDF15 expression in CRC by GCN5-mediated H3K9 acetylation

**Fig. 5:**
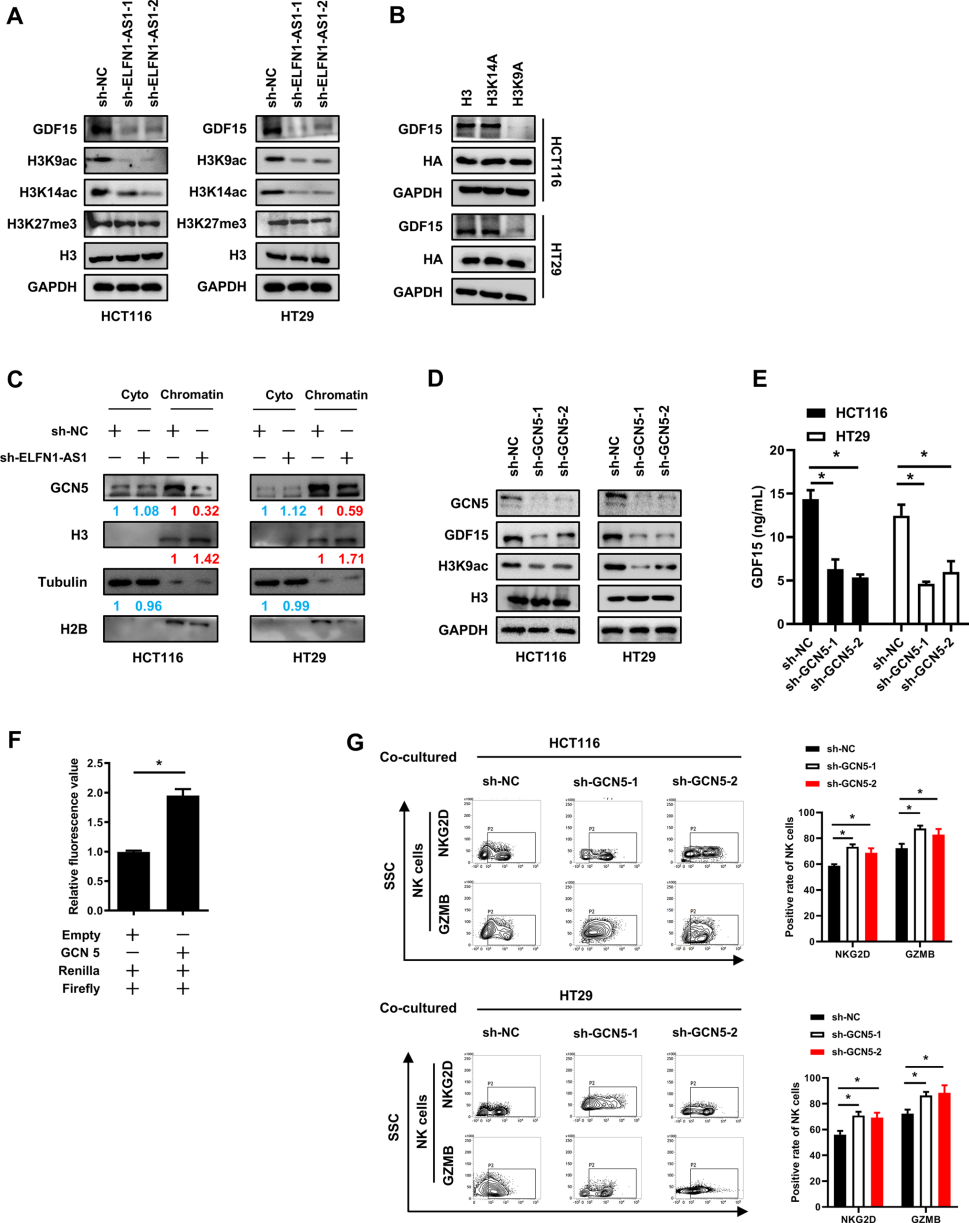
ELFN1-AS1 regulates GDF15 expression in CRC via GCN5-mediated H3K9 acetylation. Western blot was used to assess: **A** levels of GDF15, H3K9ac, H3K14ac and H3K27me3 in ELFN1-AS1-knockdown CRC cells; **B** levels of GDF15 in CRC cells containing H3K9A or H3K14A mutations; **C** levels of GCN5 in the cytoplasm or on chromatin in ELFN1-AS1 knockdown CRC cells; **D** Levels of GDF15, H3K9ac in GCN5-silenced CRC cells. **E** ELISA was used to detect the GDF15 in the supernatants from GCN5-silenced CRC cells. **F** The relative luciferase activity after co-transfection of plasmids. **G** Flow cytometry was used to assess the expression of NKG2D and GZMB in NK cell following their co-culture with GCN5- silenced CRC cells. All data are from at least three independent experiments, *P < 0.05 indicated a significant difference

Histone modifications, such as H3K9ac, H3K14ac and H3K27me3, play key roles in the gene regulation. For instance, GDF15 expression was reported to be regulated by H3K27me3 [29]. Paralleling with the GDF15 downregulation induced by ELFN1-AS1 silencing, the levels of H3K9ac and H3K14ac were decreased while H3K27me3 was not altered in CRC cells (Fig. 5A). Therefore, we constructed H3, H3K9A and H3K14A mutant plasmids with HA tag respectively and transferred them into CRC cells. HA tagged H3K9A significantly decreased GDF15 expression in comparison with wild-type but H3K14A had no effect (Fig. 5B). H3K9ac modification is primarily regulated by the histone acetylase GCN5, but GCN5 expression was unchanged in ELFN1-AS1-silenced CRC cells (data not shown). We further observed a reduction of GCN5 on chromatin in CRC cells when ELFN1-AS1 was knocked down (Fig. 5C). Knocked-down GCN5 expression in CRC cells by RNA interference significantly downregulated GDF15 expression as well as the H3K9ac (Fig. 5D). Similarly, GDF15 protein levels in the supernatants of GCN5-silenced CRC cells were also significantly decreased (Fig. 5E). Dual-luciferase reporter experiment showed that the relative luciferase activity of plasmid harboring promoter sequence of GDF15 was upregulated when co-transfected with Flag-GCN5 (Fig. 5F). Moreover, NKG2D and GZMB expression on NK cells were significantly increased after co-culture with the supernatant from GCN5-silenced CRC cells (Fig. 5G). Taken together, these results demonstrated that ELFN1-AS1 altered H3K9ac enrichment by regulating the recruitment of GCN5 to chromatin and this process promoted GDF15 expression in CRC cells.

### ELFN1-AS1 mediates GCN5-SND1 interaction leading to the alteration of H3K9ac in CRC cells

**Fig. 6:**
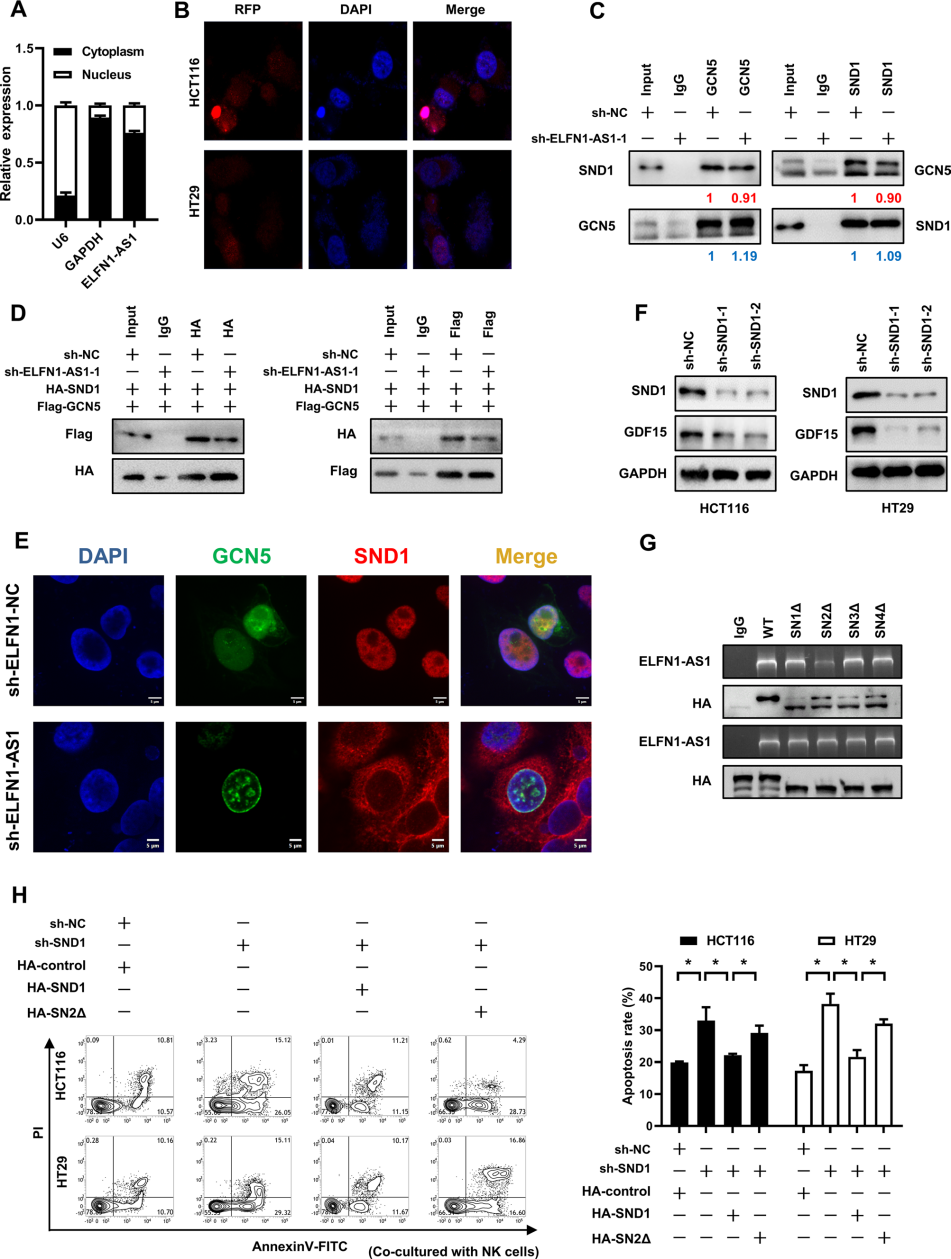
ELFN1-AS1 mediate the interaction of GCN5 and SND1 leading to the alteration of H3K9ac in CRC cells. **A** qRT-PCR was used to assess the cytoplasmic and nuclear distribution of ELFN1-AS1. **B** Immunofluorescence staining of ELFN1-AS1 in HCT116 and HT29 cells. Co-IP was used to determine: **C** the interactions between endogenous GCN5 and SND1 in ELFN1-AS1 knockdown HCT116 cells; **D** the interactions between exogenous GCN5 and SND1 in ELFN1-AS1-silenced HCT116 cells. **E** Immunofluorescence staining of GCN5 and SND1 in ELFN1-AS1-silenced HCT116 cells. **F** Western blot was used to assess the levels of GDF15 in SND1-silenced CRC cells. **G** RIP was used to determine the interaction of ELFN1-AS1 and different domain truncations of SND1 proteins. **H** Co-culture of wild type SND1 or SN2∆ truncations with NK cells influenced apoptosis of HCT116 and HT29 cells containing SND1 knockdowns. All data are from at least three independent experiments, *P < 0.05 indicated a significant difference

ELFN1-AS1 was present in both the nucleus and cytoplasm of CRC cells but higher levels were found in the cytoplasm (Fig. 6A), this was further confirmed using immunofluorescence tracking of ELFN1-AS1 in HCT116 and HT29 cells (Fig. 6B). Interestingly, there is convinced evidence that GCN5 could be recruited by SND1 to chromatin [30]. Therefore, we measured interactions between endogenous GCN5 and SND1 and in ELFN1-AS1-silenced CRC cells, this interaction was attenuated (Fig. 6C). Furthermore, immunoprecipitation assays revealed an impaired interaction between exogenous GCN5 and SND1 in ELFN1-AS1-silenced CRC cells (Fig. 6D). Moreover, we observed a colocalization of GCN5 and SND1 in CRC cells with ELFN1-AS1 silence or not (Fig. 6E). As well as GCN5 silencing, expression of GDF15 was significantly decreased by SND1 silencing in HCT116 and HT29 cells (Fig. 6F). Based on evidence that phosphorylated SND1 would enter the nucleus from the cytoplasm, we determined whether ELFN1-AS1 could directly bind to SND1 and at which domain. RNA binding protein immunoprecipitation assays revealed that the ELFN1-AS1 binding sites on SND1 were localized to the SN2 domain (Fig. 6G). We also found an increased apoptosis in SND1-silenced CRC cells induced by NK cells. Wild type SND1, but not SN2Δ plasmid transfections successfully rescued the NK cell-induced apoptosis of CRC cells (Fig. 6H). Taken together, these results indicated that ELFN1-AS1 mediated the GCN5-SND1 interaction in CRC cells via binding the SN2 domain of SND1, resulting in increased GDF15 levels in CRC cells.

## Discussion

LncRNAs are involved in multiple physiological and pathological processes, the underlying mechanisms remain largely unknown [31–33]. In this study, data showed that ELFN1-AS1 was significantly up-regulated in CRC tissues and promoted immune escape of CRC cells from NK cells. GDF15 is secreted by CRC cells and was one of the key mediators of NK cell activity. We also determined that ELFN1-AS1 regulated the expression of GDF15 through the SND1-GCN5/GDF15 axis. These results indicated that ELFN1-AS1 plays an oncogenic role in CRC progression.

Escape from immune surveillance is a pivotal feature of tumors with distant metastasis [34–36] and activation of immune surveillance is an important strategy for tumor targeted therapy. NK cells play important roles in immune surveillance and can directly kill tumor cells via perforin and granzyme release [37, 38]. There is increasing evidence that the numbers of infiltrating NK cells in tumor tissues are positively correlated with tumor patient survival [39–42]. Consistent with previous research, from TCGA data analysis we found that activated NK cell levels are decreased and resting NK cell levels are increased in CRC tissues, suggesting an altered proportion of NK cell subsets. In addition, a high proportion of resting NK cells also significantly correlated with the poor survival rate of CRC patients. These data indicated that the alteration of NK cell immunity induced by CRC tumor microenvironment may be a major mechanism for tumors to escape immune killing.

Recent studies reported that lncRNAs could regulate immune surveillance [43, 44]. Here we used gene microarrays and identified a specific lncRNA ELFN1-AS1 that was up-regulated in both CRC tissues and cells. ELFN1-AS1 has been linked to the development of multiple tumors such as esophageal [18] and ovarian [17] cancers. In colorectal cancer, ELFN1-AS1 expression was increased and promoted the proliferation and metastasis of tumor cells [21]. MYC-regulated ELFN1-AS may function in cell proliferation and the cell cycle by regulating MYC target genes [45]. Tumor immunity studies also demonstrated that some lncRNAs induce immune cell dysfunction within the tumor microenvironment [46]. Notably, in this study, we found that ELFN1-AS1 tended to be negatively associated with the surface marker CD56 on NK cells in COAD and READ, which suggested the upregulated ELFN1-AS1 may contribute to NK cell suppression. Both in vivo and in vitro, the NK cell cytotoxicity was impaired after co-culture with high level ELFN1-AS1-expressing CRC cells, implying ELFN1-AS1 could promote the immune escape of CRC cells from NK cells. Moreover, we found that the NKG2D and GZMB receptors on NK cells were significantly downregulated and the JNK signaling in NK cells was inhibited after co-culture with high level ELFN1-AS1-expressing CRC cells. JNK signaling is involved in the development and differentiation of immune cells [47]. NKG2D in NK cells can be activated by JNK signaling [48] and elevated NKG2D in turn induces activation of JNK kinase [49] and gradually activates JNK signaling pathways [50]. Inhibition of JNK MAP kinase also blocks granzyme B movement to the immune synapse [51] and the JNK pathway controls expression of CCL5 that is co-released with granzymes in NK cells [52]. Collectively, our results and previous reports suggested that ELFN1-AS1 in CRC cells might directly affect JNK signaling in NK cells to suppress the surface expression of NKG2D and GZMB resulting in a marked deficiency in tumor cytotoxicity.

Previous studies have demonstrated that tumor cells secrete NK cell inhibitory factors such as TGF-β1 and cytokines [53, 54]. In our study, we verified that the level of GDF15 (a secretory ligand of the TGF-β superfamily) was regulated by ELFN1-AS1 in CRC cells. In cervical cancer cells, GDF15 directly promoted cell proliferation and significantly increased cell cycle progression [55]. It is associated with human NK cell dysfunction that leads to the immune escape of cancers [56, 57], as well as TGF-β [58]. In addition, GDF15 is a MYC target and a positive feedback of GDF15/MYC/GDF15 was also verified [59]. Combined with the role of ELFN1-AS1 in MYC-regulated cell phenotypes, we considered that GDF15 was the secretory protein induced by ELFN1-AS1 from CRC cells. Our data also demonstrated that anti-GDF15 antibody could reverse the inhibition of NK cells induced by high ELFN1-AS1 expressed CRC cells via restoring the activity of NKG2D and GZMB in NK cells. This suggested that GDF15 production was an important mechanism used by ELFN1-AS1 to modulate CRC tumor cells to avoid NK cell cytotoxicity.

We also explored biological regulation networks between ELFN1-AS1 and GDF15 in CRC. Additional reports also indicated that EZH2 could impact GDF15 expression via H3K27me3, suggesting that histone modifications are involved in GDF15 regulation [60]. Histone modifications play a pivotal role in gene expression: H3K9ac has been correlated to active enhancers, H3K18ac is generally associated with active gene expression, and H3K27me3 was negatively correlated with transcript levels [61]. Our data indicated that ELFN1-AS1 regulated GDF15 primarily via the H3K9ac modification and not H3K14ac or H3K27me3. Histone acetylation promotes transcription by relaxing chromatin [62], H3K9ac is regulated by the GCN5-SND1 complex and contributes to cancer development [63]. In this process, GCN5 is recruited to the promoter regions to increase chromatin accessibility and acetylates H3 on the chromatin around double-strand breaks (DSB) [64]. Meanwhile, SND1 interacts with GCN5 and plays a role as a recruiter and coactivator [63, 65]. Our data verified that silencing of ELFN1-AS1 attenuated the enrichment of GCN5 on chromatin in CRC cells, leading a decrease of H3K9ac enrichment. SND1 is primarily located in the cytoplasm and is translocated into the nucleus following phosphorylation to form the GCN5-SND1 complex. Here, we identified an interaction between ELFN1-AS1 and SND1. The human SND1 protein contains 4 repeated staphylococcal nuclease-like domains (SN1 to 4) and the downstream TSN domain (Tudor plus SN5 fragments) were identified in the human SND1 protein. Consistent with this, our data demonstrated that the SN2 domain mediated the binding between SND1 and ELFN1-AS1, which linked to GDF15 expression. Moreover, as expected, SND1 silencing in CRC cells directly downregulated GDF15 secretion and co-culture with the SND1 silenced CRC cells restored the cytotoxicity of NK cells against CRC cells. Silencing of GCN5 had similar effects, indicating that ELFN1-AS1 may mediate the production of GDF15 though the GCN5-SND1 complex. GEPIA2 results also demonstrated that the expression of GCN5, SND1 and GDF15 was respectively correlated with ELFN1-AS1, (Fig. S4A-C) and this finding corroborates with our direct experimental observations.

We also analyzed the expression and regulation network of ELFN1-AS1 using RNA microarrays. HPA RNA-seq normal tissues analysis from lncbook indicated that ELFN1-AS1 was highly expressed in brain, rectum and stomach tissues (Fig. S4D). In contrast, in lncexpdb, elevated expression of ELFN1-AS1 was found in stomach, rectum and colon normal tissues (Fig. S4E). ELFN1-AS1 levels in ENCODE primary cell lines indicated a maximum transcripts per million (TPM) in kidney epithelial cells (Fig. S4F) indicating a different expression profile of ELFN1-AS1 in multiple tissues and cells. Methylation analysis also indicated that the methylation levels of promoter (Fig. S5A) and body (Fig. S5B) regions of ELFN1-AS1 are both aberrant in CRC and READ compared with normal tissues. Expression of ELFN1-AS1 is also associated with sample type (Fig. S5C-D). These suggested that aberrant methylation might be responsible for the high expression of ELFN1-AS1 in CRC tissues. Co-expression and KEGG pathway analysis revealed that ELFN1-AS1 was involved in the metabolic pathways and pathways in cancer (Fig. S6A-D), suggesting a major biological function for ELFN1-AS1 in the metabolism of CRC. All of these describe an important role for both the direct action of ELFN1-AS1 on cancer cells and an indirect action on normal cells.

Despite the significant findings in our study, several limitations remain. The present investigation mainly focused on the ELFN1-AS1/GCN5-SND1/H3K9ac/GDF15 axis, but additional mechanisms involving ELFN1-AS1 may also exist in colorectal cancer (CRC). For instance, the alteration of GCN5-SND1 cellular location may existed and also correlated with the ELFN1-AS1 levels; histone acetylation may be balanced by HAT and HDAC [66], in parallel to GCN5-SND1; Furthermore, the relationship between ELFN1-AS1 and histological type, stage, and RAS/RAF-MSI of CRC, as well as whether ELFN1-AS1 promote long-range chromatin looping as CCAT1-L in the activation of MYC [67], will be investigated in future studies. For further investigation, we will also consider more diverse metastasis models such as the CRC liver metastasis model using intra-splenic injection [68].

## Conclusions

In summary, this study describes for the first time the role of ELFN1-AS1 in the escape of CRC cells from NK killing. Overexpression of ELFN1-AS1 promoted the survival of CRC cells when encountering NK cell attacks both in vitro and in vivo. Furthermore, ELFN1-AS1 regulated the immune escape of CRC cells primarily via GDF15 that inhibited NKG2D and GZMB expression through the JNK signaling pathway in NK cells. Mechanistically, ELFN1-AS1 mediated the formation of the GCN5/SND1 complex and enhanced H3K9ac enrichment at the GDF15 promoter to reinforce transcription (Fig. S7). Our findings elucidate a potential mechanism underlying the oncogenic role of ELFN1-AS1 in CRC and indicate that ELFN1-AS1 could be a potential target, especially in CRC immune therapy.

## Supplementary Information

Supplementary file1

Supplementary file2

Supplementary file3

Supplementary file4

Supplementary file5

Supplementary file6

Supplementary file7

Supplementary file8

## Data Availability

The datasets generated during and/or analysed during the current study are available from the corresponding author on reasonable request.
