# Supplementary figures and images for: ELFN1-AS1 promotes GDF15-mediated immune escape of colorectal cancer from NK cells by facilitating GCN5 and SND1 association
Source: Discov Oncol. 2023 May 6;14:56. doi: 10.1007/s12672-023-00675-6 (PMC10163203; doi:10.1007/s12672-023-00675-6)

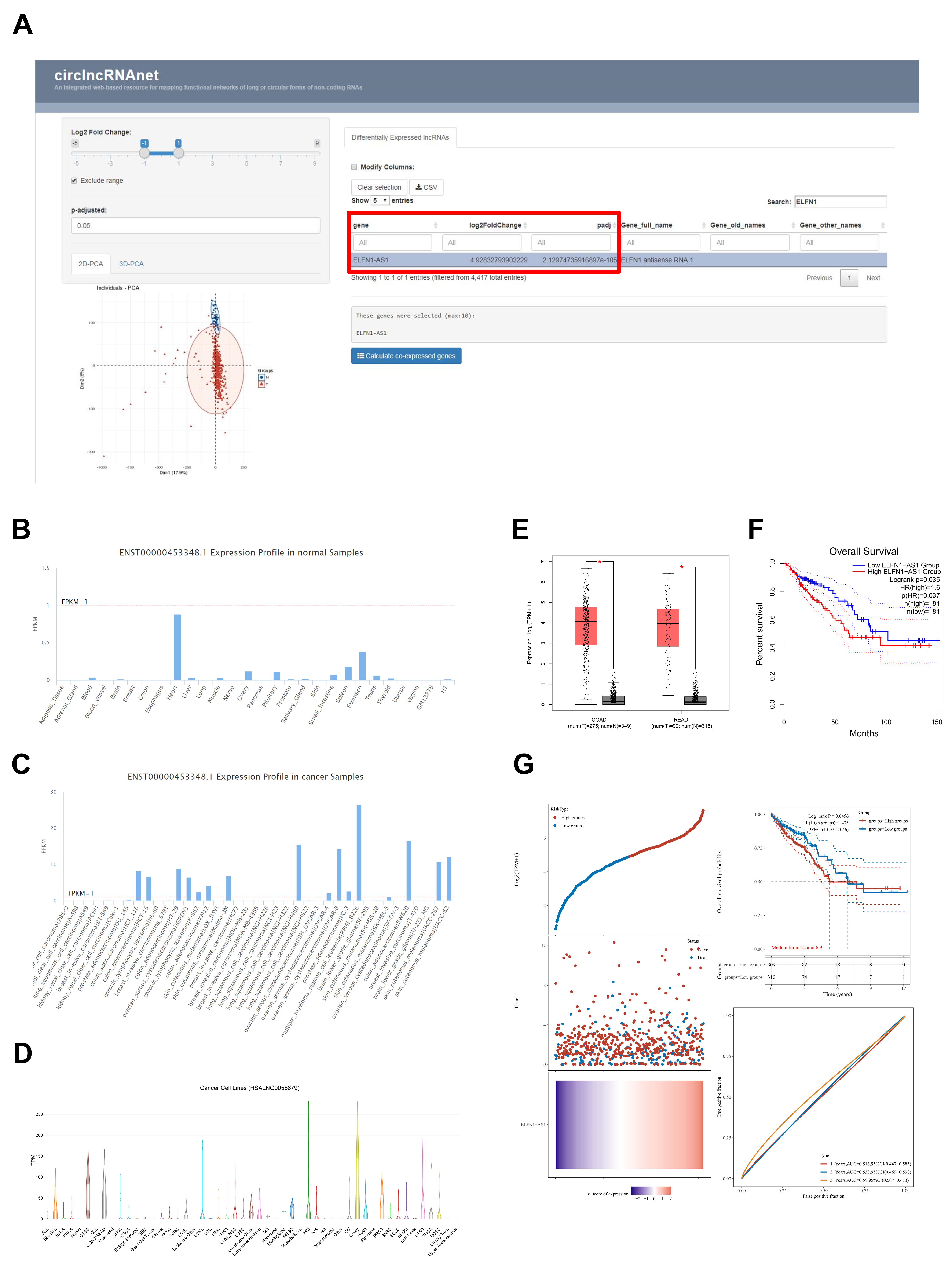

Supplement: Supplementary file 1 — Supplementary file1 [file 12672_2023_675_MOESM1_ESM.tif]

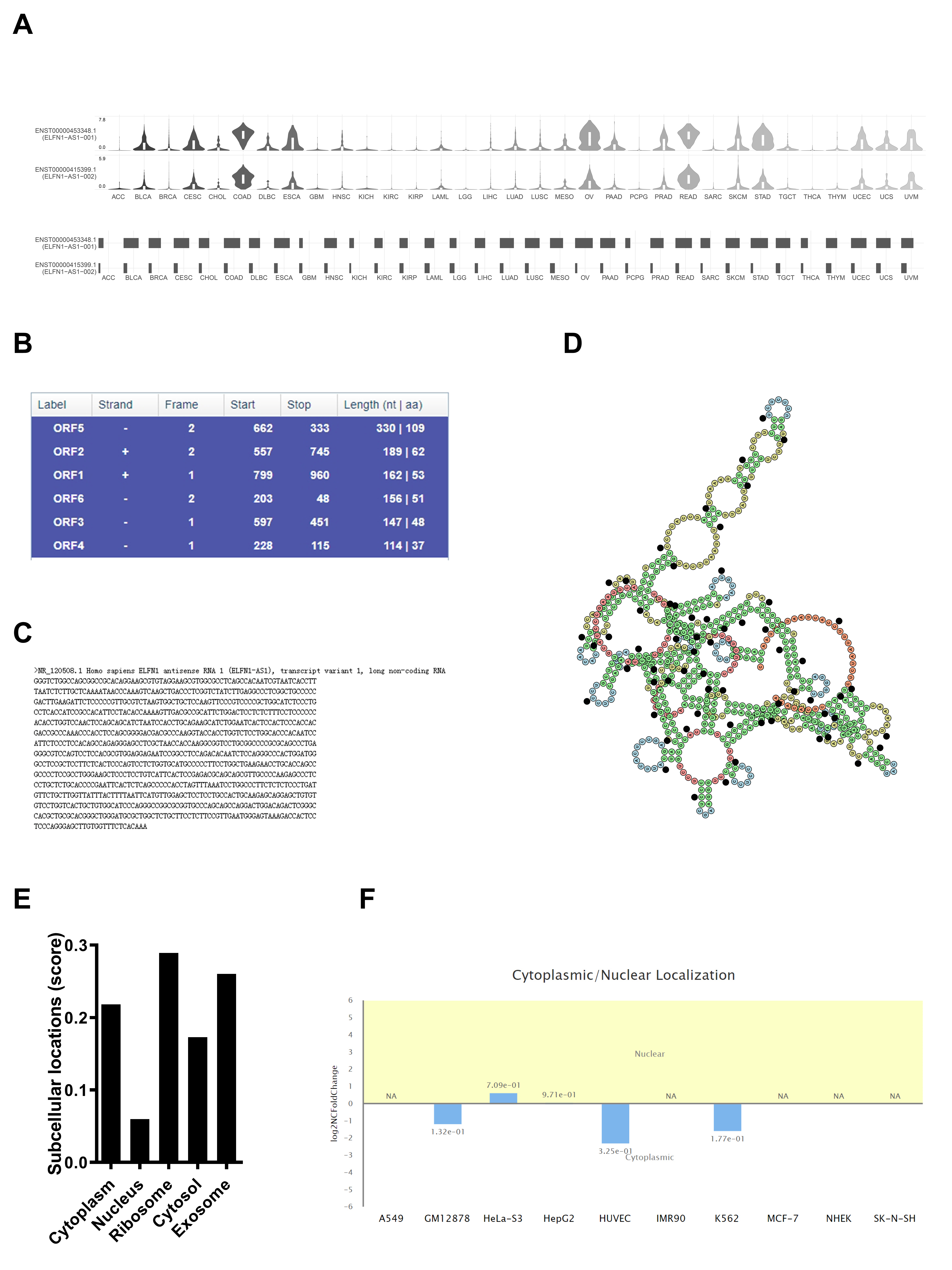

Supplement: Supplementary file 2 — Supplementary file2 [file 12672_2023_675_MOESM2_ESM.tif]

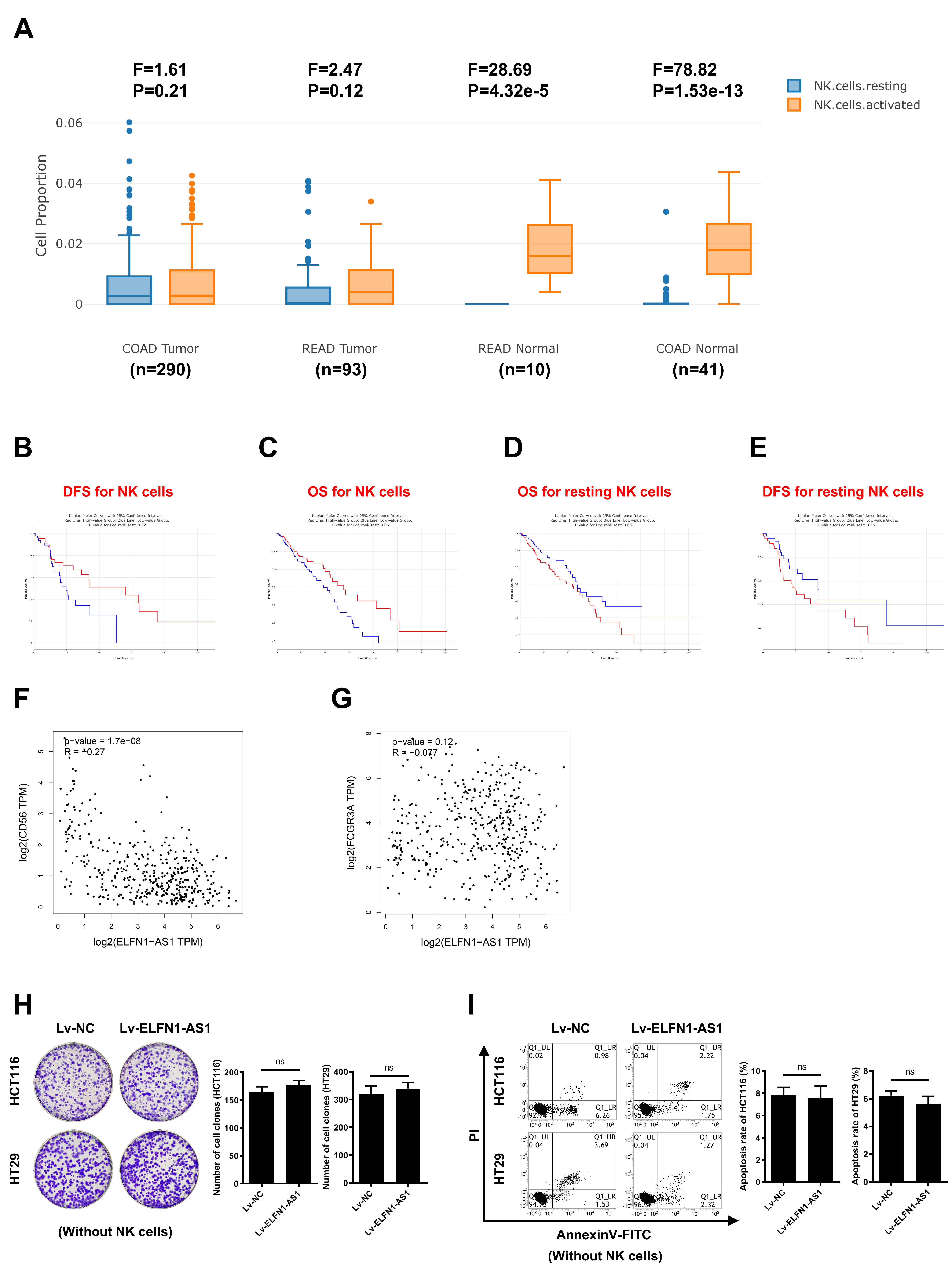

Supplement: Supplementary file 3 — Supplementary file3 [file 12672_2023_675_MOESM3_ESM.tif]

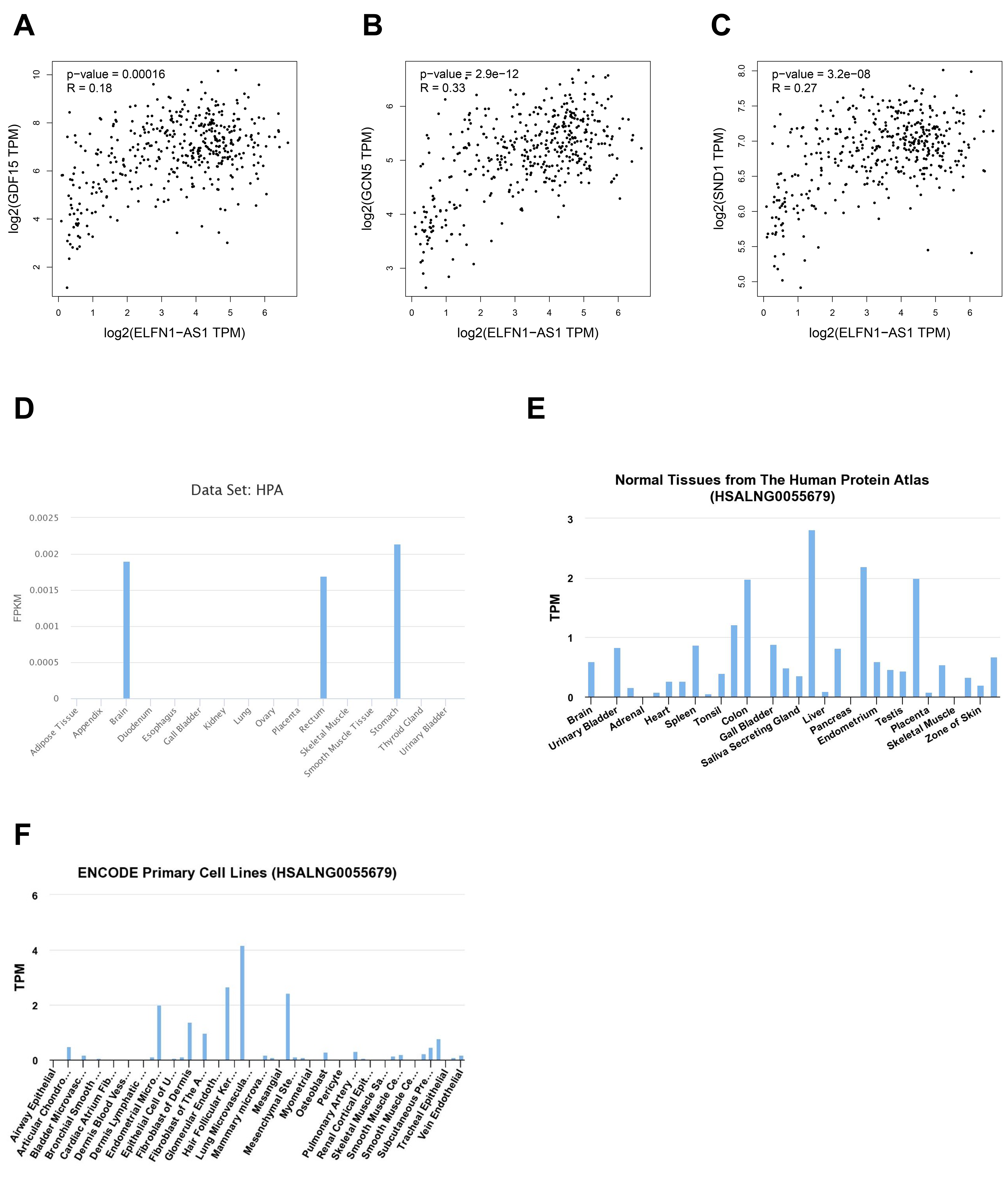

Supplement: Supplementary file 4 — Supplementary file4 [file 12672_2023_675_MOESM4_ESM.tif]

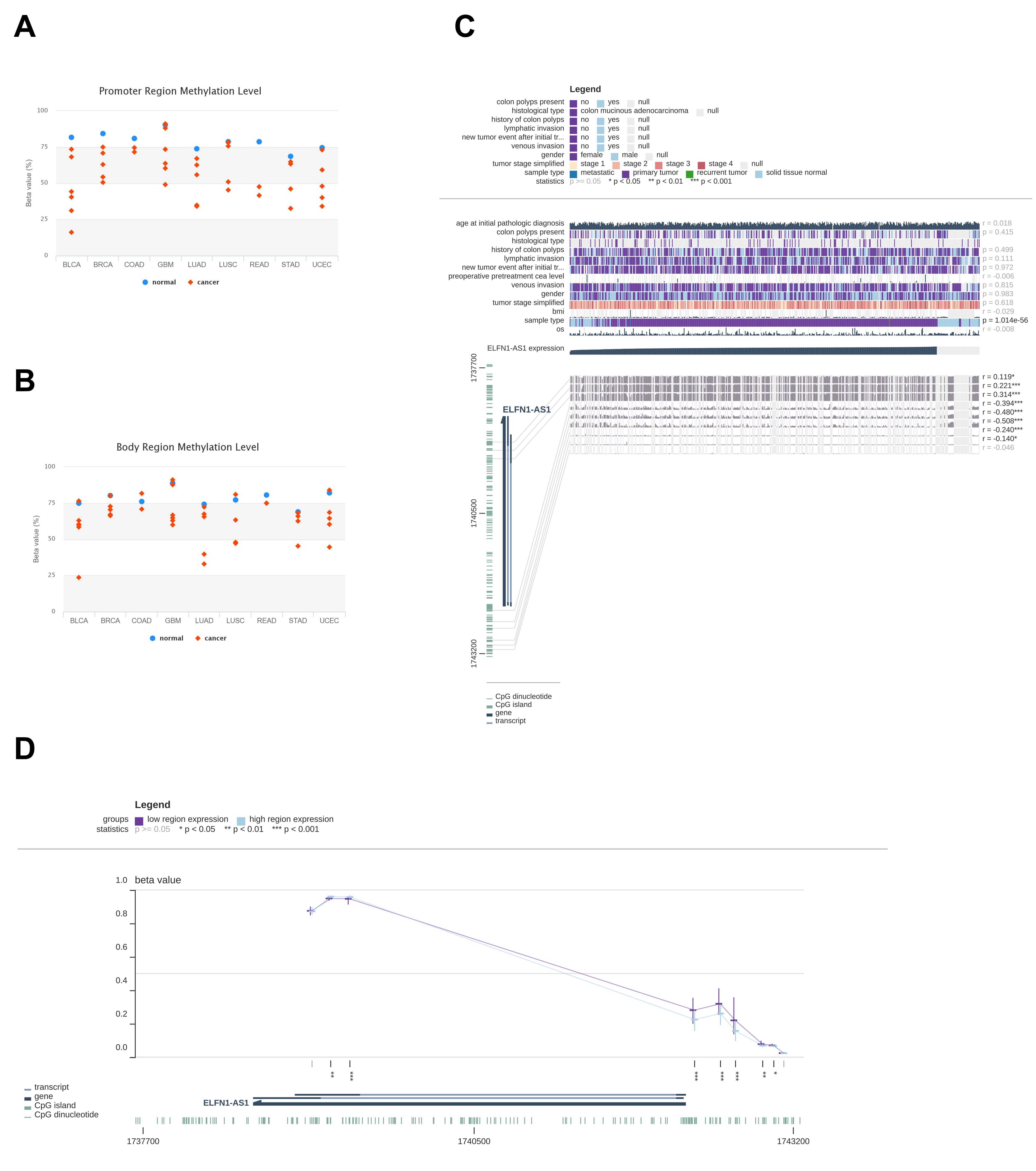

Supplement: Supplementary file 5 — Supplementary file5 [file 12672_2023_675_MOESM5_ESM.tif]

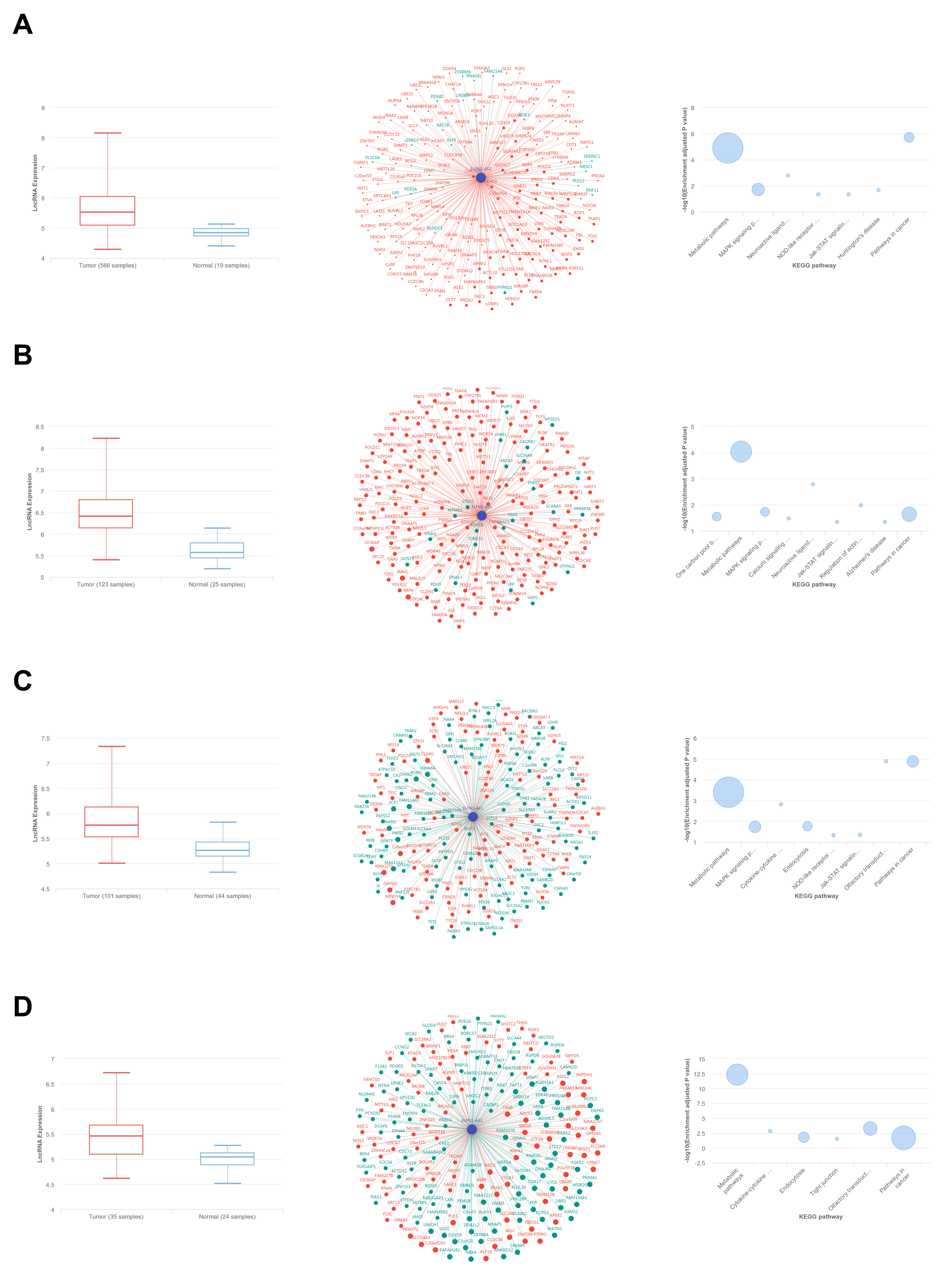

Supplement: Supplementary file 6 — Supplementary file6 [file 12672_2023_675_MOESM6_ESM.tif]

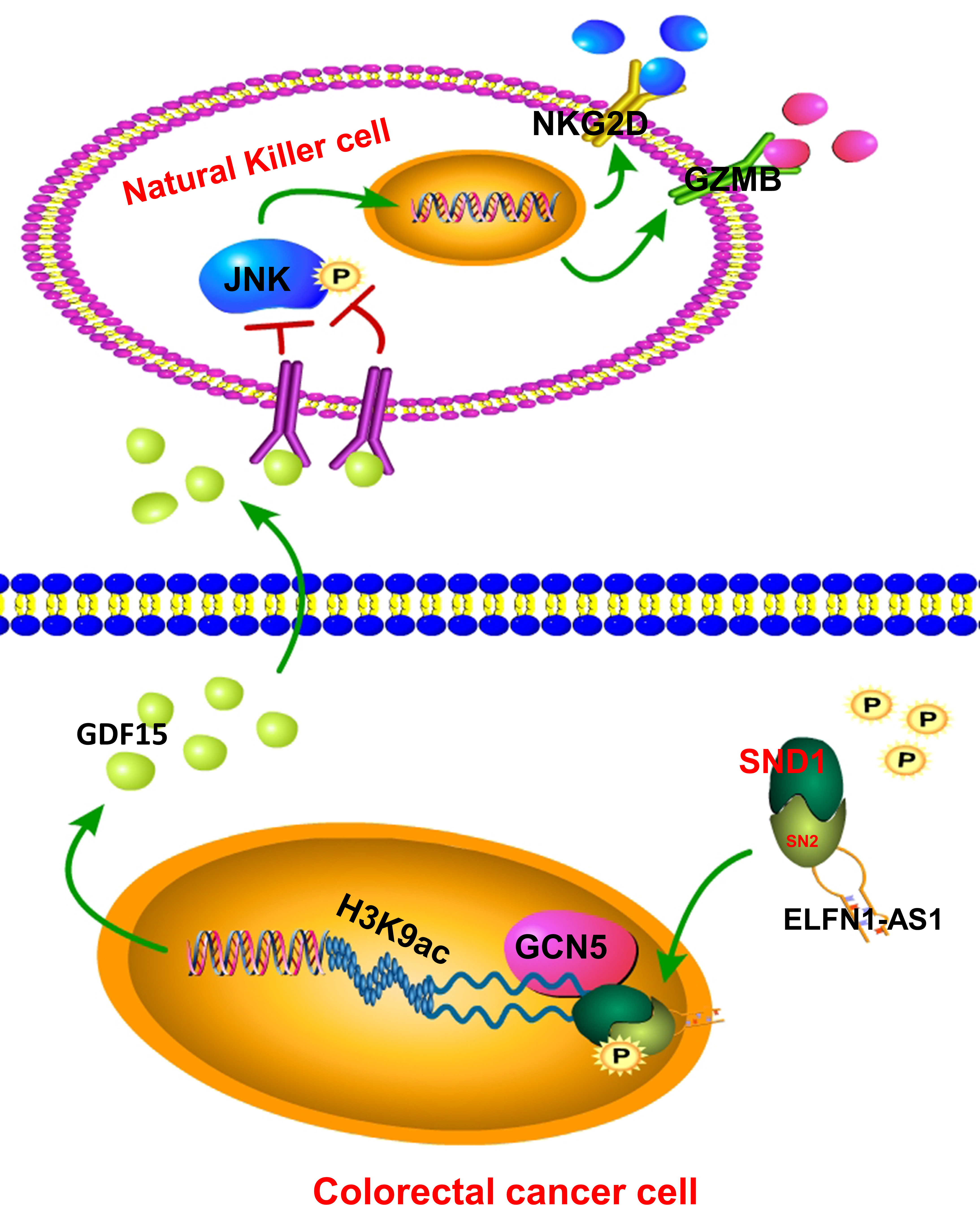

Supplement: Supplementary file 7 — Supplementary file7 [file 12672_2023_675_MOESM7_ESM.tif]
