# Supplementary material for: ELFN1-AS1 promotes GDF15-mediated immune escape of colorectal cancer from NK cells by facilitating GCN5 and SND1 association
Source: Discov Oncol. 2023 May 6;14:56. doi: 10.1007/s12672-023-00675-6 (PMC10163203; doi:10.1007/s12672-023-00675-6)
Supplement: Supplementary file 8 — Supplementary file8 [file 12672_2023_675_MOESM8_ESM.docx]

**SUPPLEMENTARY INFORMATION**

**1. Materials and methods**

**Bioinformatics**

**CirclncRNAnet** (http://120.126.1.61/circlnc/circlncRNAnet/lncRNA_TCGA/index.php) is an integrated web-based resource for mapping functional networks of long or circular forms of non-coding RNAs [1]. Long non-coding RNA (LncRNA) expression profiles were analyzed using the lncRNA-TCGA module of circlncRNAnet. The 500 most correlated co-expressed genes for ELFN1-AS1 were screened and scatter plots of correlations between gene and ELFN1-AS1 were displayed in the “co-expressed Genes (Heatmaps)” module. **AnnoLnc2** (http://annolnc.gao-lab.org/index.php) is a portal to systematically annotate novel lncRNAs for human and mouse [2] and was used to analyze ELFN1-AS1 expression in human normal and cancer samples (tissues and cells). Cytoplasmic/nuclear location analysis of ELFN1-AS1 in different cell lines was conducted by the “Subcellular Localization” module of Annolnc2. **LncExpDB** (https://ngdc.cncb.ac.cn/lncexpdb/) is a comprehensive database for lncRNA expression. It covers expression profiles of lncRNA genes across various biological contexts [3] and ELFN1-AS1 expression in different cancer cell lines were obtained from the “genes” module. **GEPIA2** (http://gepia2.cancer-pku.cn/#survival) is an updated version of GEPIA for analyzing the RNA sequencing expression of tumors and normal samples from the TCGA and the GTEx projects [4]. We used the “Expression DIY” and “Survival Analysis” to plot the expression profiles and survival curves. The expression distribution (violin plot) and isoform usage (bar plot) of ELFN1-AS1 in multiple cancer types were obtained from the Isoform Details module of GEPIA2. (http://gepia2.cancer-pku.cn/#index). Based on TCGA data, **ACLBI** (https://www.aclbi.com/) was used to analyze the prognostic stratification effect of a single gene in a sample and to examine the prognostic / gene relationships among multiple samples. A prognostic model of multiple gene signatures or the addition of clinical variables was used to construct a nomogram prognostic model and a 1, 3, 5-year receiver operating characteristic for ELFN1-AS1 in CRC. **ORF finder** (https://www.ncbi.nlm.nih.gov/orffinder/) was used to search for open reading frames (ORFs) in the ELFN1-AS1 sequence. The secondary structure of ELFN1-AS1 was displayed in the **lnCAR** (https://lncar.renlab.org/). The sequence of ELFN1-AS1 was obtained from National Center for Biotechnology Information (**NCBI**, https://www.ncbi.nlm.nih.gov/). The subcellular locations estimation was performed by the “**lncLocator**” online service from Pattern Recognition and Bioinformatics Group (http://www.csbio.sjtu.edu.cn). **GEPIA2021** (http://gepia2021.cancer-pku.cn/) allows multiple deconvolution-based analysis for GEPIA and included the sample tool in TCGA/GTEx with the bioinformatics tools CIBERSORT, EPIC and quanTIseq [5]. “Cell Type Proportion Analysis” and “Cell Type-level Survival Analysis” modules were used to analyze the natural killer (NK) cells proportion in tumors and normal samples as well as relationships between NK cells and survival. The expression levels of ELFN1-AS1 in human tissues from HPA; the promoter and body region methylation level in human cancer types were obtained from **LncBook1.0** (https://ngdc.cncb.ac.cn/lncbookv1/index), a curated knowledgebase of human lncRNAs that features a comprehensive collection of human lncRNAs. **MEXPRESS** (https://mexpress.be/) was used to summary and visualize the ELFN1-AS1 expression, methylation and clinical data, as well as the relationships between them. The expression, co-expression network and KEGG pathway of ELFN1-AS1 in colorectal cancer were obtained from the “Differential Expression Analysis” module of **lnCAR** (https://lncar.renlab.org/).

**Cell culture**

Human CRC cell lines (HCT116 and HT29) and HEK293T cells were purchased from American Type Culture Collection (ATCC, USA). Cells were cultured with Dulbecco’s modifed eagle medium (DMEM) high glucose medium (HyClone, Logan, UT, USA) supplemented with 10% fetal bovine serum (Shengong, Shanghai, China) and 1% penicillin-streptomycin solution (HyClone, Logan, UT, USA) at 37℃ in an atmosphere of 5% CO_2_.

**Plasmid construction and transfection**

The sequence of human H3.1 was amplified from the cDNA of HEK293T cells and subcloned to pLv-N-HA-empty vector. H3.1 plasmids harboring K9A and K14A mutations were generated by PCR with the indicated primers. The shRNAs for ELFN1-AS1, GCN5 and SND1 was synthetized by Sangon Biotech (Shanghai, China) and subcloned into the pLKO.1-TRC plasmid harboring the U6 promotor. All the plasmids were sequenced and validated by Sangon. Primers and shRNA sequences are listed in Table S1. Lipofectamine 3000 (Invitrogen, USA) was used for oligonucleotide transfection according to the manufacturer’s protocol. For ELFN1-AS1 ectopic expression, the sequence of ELFN1-AS1 was amplified from cDNA of HEK293T and subcloned into pcDH-CMV-MCS-EF1 plasmid digested by EcoRI and BamHI followed by sequencing. The endo-free plasmids were isolated using an E.Z.N.A. Endo-Free Plasmid DNA Midi kit (Omega Bio-Tek, USA) according to the manufacturer’s protocol. To obtain indicated viral particles, viral plasmids were then co-transfected into HEK293T cells with pMD2.G and psPAX.2 in the ratio of 3:2:1. The viral particles were collected at 72h and frozen at -80°C after filtration with 0.22 µm filters.

**RNA extraction, qRT-PCR, ELISA and Western blot assays**

RNA extraction, qRT-PCR, and Western blot assays were performed as described previously [6]. Nuclear and cytoplasmic RNA were isolated separately using the Cytoplasmic & Nuclear RNA purification kit (Norgen Biotek, Thorold, ON, USA) according to the manufacturer's protocol. Protein from cell cytosol and Chromatin was extracted using the Nuclear/Cytosol Fractionation Kit according to the manufacturer's protocol. For qRT-PCR, gene expression was normalized using the 2^-⊿⊿^*C*_t_ method from the *C*_t_ values of interest relative to GAPDH. Human GDF-15 ELISA Kit (Abcam) was used to detect the protein content of GDF15 in cell culture supernatant according to the manufacturer's protocol.

**Cell proliferation**

HCT116 and HT29 cells were seeded at a density of 1 × 10^3^ cells per well in 24-well plates and cultured overnight. The NK cells were co-cultured with CRC cells at a ratio of 10: 1 for 12h (CRC cells without NK cells treatment omitted the co-culture step). The NK cells were removed and cultured was continued for 10 d and the CRC cells were rinsed with PBS, fixed in 4% paraformaldehyde and stained with 5% crystal violet for 30 min. The colonies were characterized and counted for the evaluation of cell proliferation.

**Cell apoptosis**

The CRC cells were co-cultured with the NK cells at a ratio of 1: 10 for 12h (CRC cells without NK cells treatment omitted the co-culture step). After removing the NK cells, AnnexinV-FITC / PI double-staining was used to detect apoptosis as described previously [6].

**NK cell surface markers detection**

The supernatants of conditioned CRC cells were collected and co-cultured with NK cells for 24 h. NK cells were stained with indicated fluorescent antibodies for related surface markers (NKG2D, GZMB, NKp30, NKp44, NKp46, CD16, NKG2A and CD158b). The gating strategy of NK cells was as follows: for example, first we gated the NK cells by CD3-CD56+; furthermore, CD3-CD56+NK cells were gated by NKG2D+, the ratio of NKG2D+ NK cells was determined. The same strategy was used for other related marker detection (Fig. 2B). The expression levels of surface markers and effector molecules of NK cells were detected by flow cytometry using a FACSCanto II instrument (BD Biosciences). Data were analyzed with FACSDiva software (BD Biosciences) or FlowJo software (TreeStar). The fluorescent antibodies are listed in the supplementary information.

**Dual-luciferase reporter assay**

To determine the promoter region of GDF15 is regulated by the recruitment of GCN5, we performed dual-luciferase reporter assay as described previously [6]. In brief, the promoter sequence of GDF15 was amplified from DNA of HCT116 and subcloned into pGL3 plasmid, which was sequenced by Sangon Biotech. The plasmid of Flag-GCN5, pGL3 harboring promoter sequence of GDF15 and phRL-TK were co-transfected into HeLa cells. Indicated cells were lysed and mixed with substrate of firefly luciferase Lar II in the ratio of 1:4. After measuring fluorescence value, the substrate of Renilla luciferase -Stop&Glo was added into the sample to measure the values of Renilla luciferase. Finally, we calculated the ratio of Firefly luciferase/Renilla luciferase for each tube, and then used the ratio of control group as a unit of 1 to obtain the relative luciferase activity of different treatment groups.

**In Situ Hybridization and Immunofluorescence**

A customized Hybridization *in situ* kit (Boster Biological Technology Co., Ltd, China) with SABC-POD was used to detect ELFN1-AS1 expression *in situ* in the HCT116 and HT29 cell lines according to the manufacturer’s protocol. CRC cells were washed with PBS twice, following fixed by 4% paraformaldehyde for 15 min at room temperature. Then, the cells were permeabilized by PBS containing 0.5% Triton X-100 for 10 min in ice bath, following processed by the Hybridization *in situ* kit using the Ribo^TM^ lncRNA FISH Probe Mix (Red). Finally, the cells were counterstained with DAPI for 10 min and were then imaged using a confocal fluorescence microscope (Olympus, Japan)

CRC cells lines with ELFN1-AS1 silence or not were seeded in µ-Slide 8 Well ibiTreat slide chambers (Ibidi, Martinsried, Germany) and grown to 80% confluency. Then we fixed the cells with 4% paraformaldehyde for 10 min at room temperature (RT) and permeabilized them with PBS containing 0.5% Triton X-100 for 10 min at RT. The cells were then incubated with 5% BSA (blocking solution) for 2h at RT and stained for GCN5 and SND1, and cell nuclei of cells were counterstained with DAPI. Slide chambers were observed with confocal fluorescence microscope (Olympus, Japan).

**Antibodies for assays**

Primary antibodies included anti-CD3 antibody rabbit mAb (Abcam), anti-CD56 antibody rabbit mAb (Abcam), anti-vimentin rabbit mAb (Cell Signaling Technology, CST), anti-GAPDH rabbit mAb (CST), anti- E-Cadeherin mouse mAb (Abcam), anti-NKG2D antibody rabbit mAb (Abcam), anti-GZMB antibody rabbit mAb (Abcam), anti-NKp30 antibody rabbit mAb (Abcam), anti-NKp44 antibody rabbit mAb (Abcam), anti- NKp46 mouse mAb (Abcam), anti-CD16 antibody rabbit mAb (Abcam), anti-NKG2A antibody rabbit mAb (Abcam), anti-CD158b antibody rabbit mAb (Abcam), anti-phospho-JNK1 (Thr183/Tyr185) antibody rabbit mAb (Abcam), anti-JNK1 antibody rabbit mAb (Abcam), anti-GDF15 antibody rabbit mAb (Abcam), anti-Histone H3 (acetyl K9) antibody rabbit pAb (Abcam), anti-Histone H3 (acetyl K14) antibody rabbit pAb (Abcam), anti-Histone H3 (tri methyl K27) antibody rabbit pAb, anti-HA tag antibody rabbit mAb (Abcam), anti-DDDDK tag (Binds to FLAG® tag sequence) antibody mouse mAb (Abcam), anti-GCN5 antibody rabbit mAb (Abcam), anti-SND1 antibody rabbit pAb (Abcam), anti-β-Tubulin antibody rabbit mAb (Abcam), anti-RFP antibody rabbit pAb (Abcam), anti-Histone H2B antibody rabbit mAb (Abcam), anti-Histone H3 antibody rabbit mAb (Abcam), anti-IgG antibody rabbit mAb (Abcam), HRP-conjugated secondary Abs (Zhongshan Biotechnology, Beijing, China) were used.

**2. Supplemental Figure Legends**

**Fig**. **S1** ELFN1-AS1 expression is frequently increased in colorectal cancer (CRC) and is associated with the poor survival of CRC patients. **A** ELFN1-AS1 expression levels in human CRC tissues (data from circlncRNAnet). The expression profiles of ELFN1-AS1 in human normal **(B)** and cancer **(C)** samples (data from Annolnc2). **D** The expression of ELFN1-AS1 in cancer cell lines (data from LncExpDB). **E** ELFN1-AS1 expression levels in cancerous and normal tissues of COAD and READ (data from GEPIA2). **F** Association between ELFN1-AS1 expression and overall survival in patients with CRC (data from GEPIA2). **G** Prognostic analysis of NUDCD1 signature in CRC (data from ACLBI). *P<0.05, compared with normal tissues.

**Fig. S2 A** The alternatively spliced isoforms of ELFN1-AS1 in different cancers. **B** ORF Finder analysis reveals the possible open reading frames of ELFN1-AS1. The sequence **(C)** and secondary structure **(D)** of ELFN1-AS1. **E** Subcellular locations estimation of ELFN1-AS1. **F** Cytoplasmic/nuclear location analysis of ELFN1-AS1 from Annolnc2.

**Fig**. **S3** Associations between NK cells, ELFN1-AS1 and survival in CRC. **A** Proportions of resting and activated NK cells in cancerous and normal tissues of COAD and READ (data from GEPIA2021). Relationships between NK cell numbers and disease-free survival (DFS) **(B)** or overall survival (OS) **(C)** in COAD and READ (data from GEPIA2021). Relationships between the numbers of resting NK cells and OS **(D)** or DFS **(E)** in COAD and READ (data from GEPIA2021). **F** The correlation between ELFN1-AS1 and CD56 (NCAM1) in cancerous and normal tissues of COAD and READ (data from GEPIA2). **G** The correlation between ELFN1-AS1 and CD16 (FCGR3A) in cancerous and normal tissues of COAD and READ (data from GEPIA2). Without co-culture with NK cells, the cell colony formation **(H)** and apoptosis **(I)** of ELFN1-AS1-overexpressing CRC cell lines.

**Fig. S4** The relationship between ELFN1-AS1 expression and GDF15 **(A**), GCN5 **(B)**, SND1 **(C)** in COAD and READ. **D** ELFN1-AS1 expression levels in human normal tissues from HPA in lncbook. **E** ELFN1-AS1 expression levels in normal tissues from HPA in lncexpdb. **F** ELFN1-AS1 expression levels in ENCODE primary cell lines.

**Fig. S5** The promoter **(A)** and body **(B)** region methylation level of ELFN1 in different cancers. **C** The methylation, expression and clinical data for ELFN1-AS1 in COAD. **D** The summarized view of methylation, expression and clinical data for ELFN1-AS1 in COAD.

**Fig. S6** The expression, co-expression network and KEGG pathway of ELFN1-AS1 in colorectal cancer from lnCAR. **(A)** 566 tumor samples vs 19 normal samples; **(B)** 123 tumor samples vs 25 normal samples; **(C)** 101 tumor samples vs 44 normal samples; **(D)** 35 tumor samples vs 24 normal samples.

**Fig. S7** A proposed model of relationship between ELFN1-AS1 in colorectal cancer cells and cytotoxic activity of NK cells. In colorectal cancer (CRC) cells, ELFN1-AS1 mediated the formation of the GCN5/SND1 complex and enhanced H3K9ac enrichment at the GDF15 promoter to facilitate GDF15 synthesis and secretion. The tumor-derived GDF15 suppressed the expression of NKG2D and GZMB via inhibiting JNK signaling in NK cells, thus impairing the cytotoxic activity of NK cells to promote CRC cell surveillance escape.

**3. Supplemental Table**

**Table S1. The sequences of primers and shRNAs**

| ELFN1-AS1 | Sense: 5^,^-ACAGGAAGCGTGTAGGAAGC-3 |
| --- | --- |
|  | Antisense: 5^,^-AGTGAATTCGGGGTGCAGAG-3^,^ |
| GDF15 | Sense: 5′-TCACCACAACCTCTGCCTCCT-3′ |
|  | Antisense: 5′-TTGAGACCAGCCTGACCAACATG-3′ |
| GAPDH | Sense: 5′-CCTTGAACTCGGTTCTCAATTCC-3′ |
|  | Antisense: 5′- CAATGGTCTGGTACTTATTCCCG-3′ |
| U6 | Sense: 5′-TGGTCTGTGTAGTTGTGGCTGAA-3′ |
|  | Antisense: 5′-GCGTCTTCTCTTGTCTTCTGCTAT-3′ |
| β-actin | Sense: 5′-TTCCTTCCTGGGCATGGAGTCC-3′ |
|  | Antisense: 5′-TGGCGTACAGGTCTTTGCGG-3′ |
| shELFN1-AS1-1 | 5^,^- CCGG GCTAGGGCTTTGTGCATTTGA CTCGAG TCAAATGCACAAAGCCCTAGC TTTTTG -3^,^ |
| shELFN1-AS1-2 | 5^,^- CCGG GCAGTGGCACTACATGTAACC CTCGAG GGTTACATGTAGTGCCACTGC TTTTTG -3^,^ |
| sh-GCN5-1 | 5^,^-CCGG GGAGAATGTGTCAGAGGATGA CTCGAG TCATCCTCTGACACATTCTCC TTTTTG-3^,^ |
| sh-GCN5-2 | 5^,^- CCGG GGAGAATGTGTCAGAGGATGA CTCGAG TCATCCTCTGACACATTCTCC TTTTTG -3^,^ |
| sh-SND1-1 | 5^,^-CCGGGCTGATGATGCAGACGAATTTCTCGAG AAATTCGTCTGCATCATCAGC TTTTT -3^,^ |
| sh-SND1-2 | 5^,^-CCGGGAAGGCATGAGAGCTAATAATCTCGAG ATTATTAGCTCTCATGCCTTC TTTTTG -3 |

**4. References**

1. Wu SM, Liu H, Huang PJ, Chang IY, Lee CC, Yang CY, et al. circlncRNAnet: an integrated web-based resource for mapping functional networks of long or circular forms of noncoding RNAs. Gigascience. 2018;7:1-10.

2. Ke L, Yang DC, Wang Y, Ding Y, Gao G. AnnoLnc2: the one-stop portal to systematically annotate novel lncRNAs for human and mouse. Nucleic Acids Res. 2020;48:W230-W8.

3. Li Z, Liu L, Jiang S, Li Q, Feng C, Du Q, et al. LncExpDB: an expression database of human long non-coding RNAs. Nucleic Acids Res. 2021;49:D962-D8.

4. Tang Z, Kang B, Li C, Chen T, Zhang Z. GEPIA2: an enhanced web server for large-scale expression profiling and interactive analysis. Nucleic Acids Res. 2019;47:W556-W60.

5. Li C, Tang Z, Zhang W, Ye Z, Liu F. GEPIA2021: integrating multiple deconvolution-based analysis into GEPIA. Nucleic Acids Res. 2021;49:W242-W6.

6. Han B, Xu K, Feng D, Bai Y, Liu Y, Zhang Y, et al. miR-144 inhibits the IGF1R-ERK1/2 signaling pathway via NUDCD1 to suppress the proliferation and metastasis of colorectal cancer cells: a study based on bioinformatics and in vitro and in vivo verification. J Cancer Res Clin Oncol. 2022;148:1903-18.
